# Gluconeogenic enzyme PCK1 supports S-adenosylmethionine biosynthesis and promotes H3K9me3 modification to suppress hepatocellular carcinoma progression

**DOI:** 10.1172/JCI161713

**Published:** 2023-07-03

**Authors:** Dongmei Gou, Rui Liu, Xiaoqun Shan, Haijun Deng, Chang Chen, Jin Xiang, Yi Liu, Qingzhu Gao, Zhi Li, Ailong Huang, Kai Wang, Ni Tang

**Affiliations:** 1 Key Laboratory of Molecular Biology for Infectious Diseases (Ministry of Education), Institute for Viral Hepatitis, Department of Infectious Diseases, The Second Affiliated Hospital,; 2 Institute of Life Sciences, and; 3 Department of Breast and Thyroid Surgery, The Second Affiliated Hospital, Chongqing Medical University, Chongqing, China.

**Keywords:** Metabolism, Oncology, Gluconeogenesis, Liver cancer

## Abstract

Deciphering the crosstalk between metabolic reprogramming and epigenetic regulation is a promising strategy for cancer therapy. In this study, we discovered that the gluconeogenic enzyme PCK1 fueled the generation of S-adenosylmethionine (SAM) through the serine synthesis pathway. The methyltransferase SUV39H1 catalyzed SAM, which served as a methyl donor to support H3K9me3 modification, leading to the suppression of the oncogene *S100A11*. Mechanistically, PCK1 deficiency–induced oncogenic activation of S100A11 was due to its interaction with AKT1, which upregulated PI3K/AKT signaling. Intriguingly, the progression of hepatocellular carcinoma (HCC) driven by PCK1 deficiency was suppressed by SAM supplement or S100A11 KO in vivo and in vitro. These findings reveal the availability of the key metabolite SAM as a bridge connecting the gluconeogenic enzyme PCK1 and H3K9 trimethylation in attenuating HCC progression, thus suggesting a potential therapeutic strategy against HCC.

## Graphical abstract

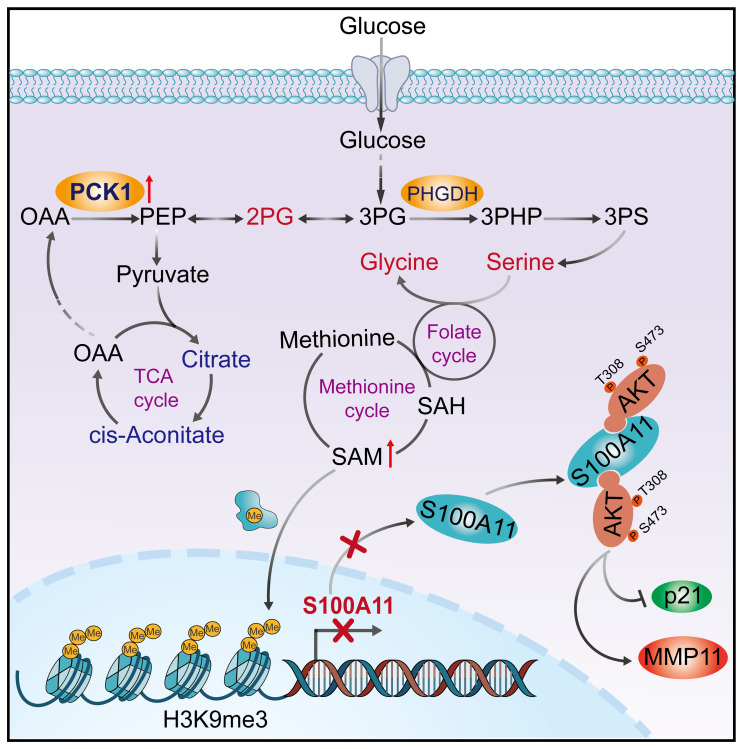

## Introduction

Altered epigenetics and aberrant gene expression patterns are the major features of cancer (1). Dysregulated posttranslational modifications of histones, such as methylation and acetylation, are believed to play important roles in the onset and progression of numerous cancers (2, 3). Histone methylation is catalyzed by histone methyltransferases (HMTs), which can either activate or repress gene expression depending on the specific histone residue that is modified as well as the number of methyl groups added (3, 4). The most extensively studied histone methylation sites, including H3K4, H3K36, and H3K79, are generally associated with transcriptional activation, while H3K9me2, H3K9me3, and H3K27me3 act as repressive marks (5–8). HMTs use S-adenosylmethionine (SAM) as the methyl donor that transfers its methyl group to yield S-adenosylhomocysteine (SAH) and a methylated substrate (9). SAM is derived from the methionine cycle via methionine adenosyl-transferases. Cellular SAM concentrations are commensurate with enzyme Km values (concentration of SAM at half-maximum velocity of the methyl transferase reaction) (10, 11), and changes in methylation status occur because of differences in the enzymatic activity of HMTs (9). Consequently, histone methylation status might be influenced by fluctuations in SAM or regulators of SAM synthesis, linking metabolism to epigenetic regulation (12). Recent studies have demonstrated that the liver can be considered as the body’s SAM factory, where nearly 85% of all methylation reactions take place (13, 14), suggesting that abnormal SAM levels or aberrant histone methylation may play important roles in the occurrence of cancer.

Gluconeogenesis, the reverse pathway of glycolysis, occurs mainly in the liver and plays key roles in metabolic reprogramming and tumor growth (15). The cytosolic isoform PCK1 (also known as PEPCK-C or PEPCK1), the initial enzyme in hepatic gluconeogenesis, catalyzes the conversion of oxaloacetate (OAA) to phosphoenolpyruvate (PEP) (16). Previous studies have found that PCK1 is downregulated in hepatocellular carcinoma (HCC), and KO of PCK1 enhances the proliferation and metastasis of HCC in vitro and in vivo (17–21). Metabolic reprogramming by PCK1 can promote cataplerosis in the tricarboxylic acid (TCA) cycle (18), disrupt hexosamine-biosynthesis pathway–mediated O-GlcNAcylation(21), and regulate T cell memory through the pentose phosphate pathway (22). However, the complex metabolic functions and mechanisms of PCK1 underlying HCC have not been well defined, and whether PCK1 plays a metabolic role in histone methylation to control gene expression remains unknown.

Here, we detected the relative levels of various histone methylation markers and found that histone H3 lysine 9 trimethylation (H3K9me3) was substantially decreased in PCK1-deficient HCC cells and the liver tissues of hepatocyte-specific *Pck1*-KO (LKO) mice. Moreover, metabolomic data showed that the serine synthesis pathway (SSP) is markedly upregulated in PCK1-overexpressing (PCK1-OE) hepatoma cells. Serine facilitates 1-carbon metabolism, which comprises a network of interconnected metabolic pathways that promote the utilization of 1-carbon units for biosynthesis of SAM (23, 24). Our data imply that PCK1 depends on the diversion of TCA intermediates into the SSP, modulating the availability of SAM to support the repressive mark H3K9me3 at the promoter of oncogene *S100A11*, thus attenuating the expression of *S100A11*. Our findings reveal that PCK1 controls SAM availability and allows direct communication between metabolic signaling and the epigenetic state, potentiating approaches to clinical applications to treat HCC.

## Results

### PCK1 upregulates H3K9me3 levels and provides methyl donors by enhancing SSP flux.

**Figure 1:**
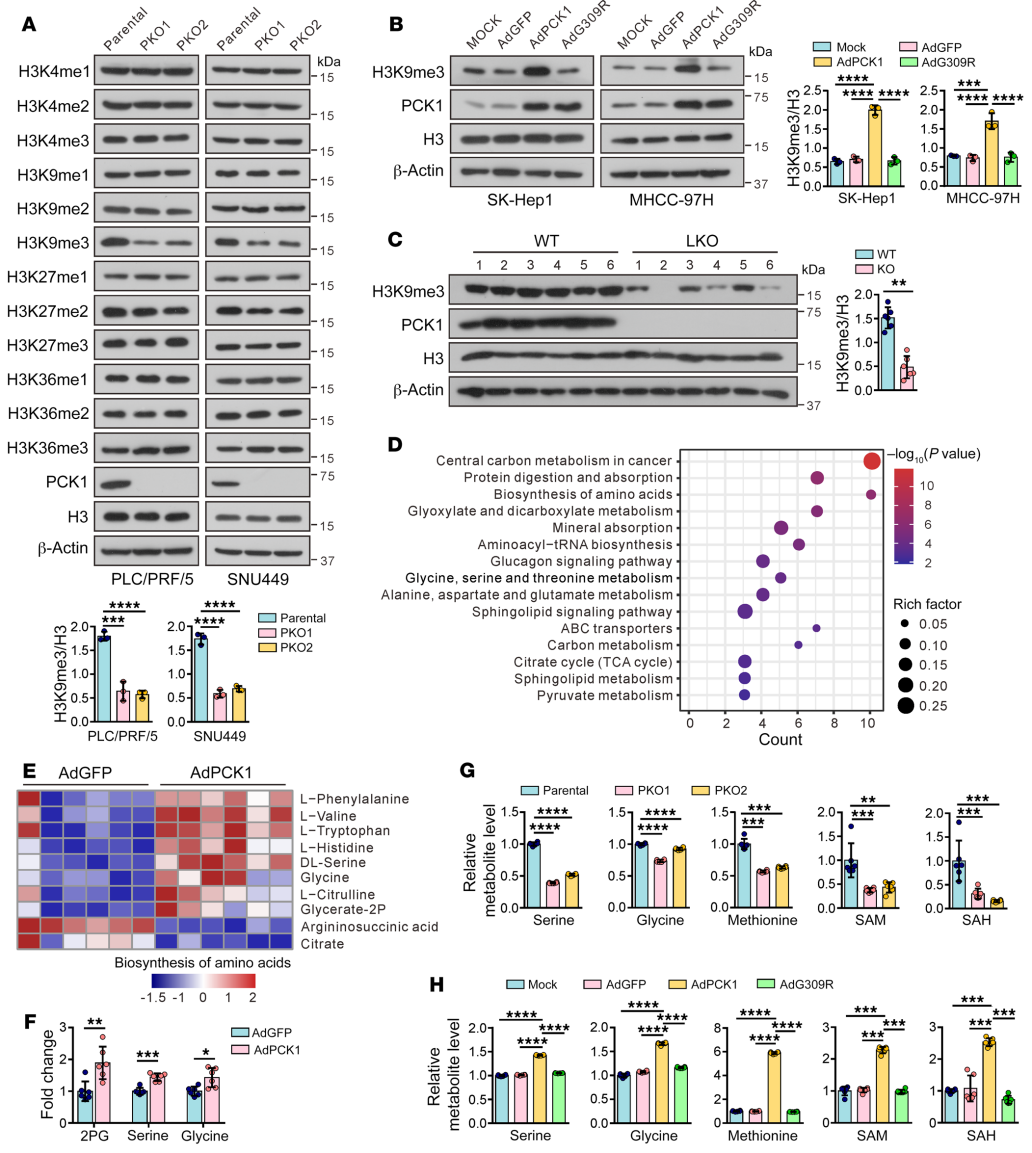
PCK1 upregulates H3K9me3 levels and provides methyl donors by enhancing SSP flux. (**A**) Immunoblots of the indicated proteins or histone modification markers in PCK1-KO PLC/PRF/5 cells (PKO cells) and PCK1-KO SNU449 cells (PKO cells). Data from 1 representative experiment are shown (*n* = 3). (**B**) Western blots from SK-Hep1 cells and MHCC97H cells overexpressing GFP (control cells), WT PCK1, or an enzymatically deficient mutant (PCK1 G309R). Mock-treated cells served as a blank control. Data from 1 representative experiment are shown (*n* = 3). (**C**) Immunoblots in liver tumors from DEN/CCl_4_-induced WT and LKO mice and densitometric analysis of H3K9me3 (*n* = 6 mice per group). (**D**) Enrichment bubble metabolic pathways, (**E**) heatmap showing changes in biosynthesis of amino acids, and (**F**) fold changes in intermediate metabolites of the SSP in SK-Hep1 cells overexpressing WT PCK1 (*n* = 6 biologically independent samples). 2PG, 2-phosphoglycerate. (**G** and **H**) LC-MS analysis of intracellular metabolites in (**G**) PKO cells and (**H**) PCK1-OE cells (*n* = 6 biologically independent samples). Data are shown as the mean ± SEM. Statistical analysis was performed using 2-tailed unpaired Student’s *t* test (**C** and **F**) or 1-way ANOVA with Tukey’s test (**A**, **B**, **G**, and **H**). *P < 0.05, **P < 0.01, ***P < 0.001, ****P < 0.0001.

To explore the role of the gluconeogenesis enzyme PCK1 in histone methylation, we examined histone methylation markers, including H3K4me1/me2/me3, H3K9me1/me2/me3, H3K27me1/me2/me3, and H3K36me1/me2/me3 methylation in PCK1-KO (PKO) PLC/PRF/5 cells and SNU449 cells. Loss of PCK1 largely reduced H3K9me3 and, to a lesser extent, H3K27me2 modifications. H3K4me1/me2/me3, H3K9me1/me2, H3K27me1/me3, and H3K36me1/me2 modifications were less affected by PCK1 KO, while H3K36me3 appeared to increase slightly. Quantitation of the H3K9me3 modifications indicated that reduced histone H3K9me3 was associated with PCK1 deficiency in HCC cells (Figure 1A and Supplemental Figure 1A; supplemental material available online with this article; https://doi.org/10.1172/JCI161713DS1). In addition, H3K9me3 was increased in SK-Hep1 cells and MHCC-97H cells overexpressing WT PCK1 (PCK1-OE), whereas fewer changes were noted among cells overexpressing GFP (control cells), enzymatically deficient mutant cells (PCK1 G309R), and mock-treated cells (Figure 1B). Similar to the changes in cells, immunoblots showed significant downregulation of H3K9me3 in the liver tissues of *Pck1^fl/fl^*:Alb-Cre^+/−^ LKO mice compared with WT mice (Figure 1C).

H3K9me3 is a key inactive epigenetic marker, and its dysregulation has been observed in many human cancers (8, 25, 26). We aimed to understand the molecular mechanisms underlying the positive correlation between H3K9me3 and the metabolic enzyme PCK1 during liver cancer development. An unbiased liquid chromatograph–mass spectrometry–based (LC-MS–based) metabolomic analysis revealed that PCK1 diverted TCA intermediates into the SSP in PCK1-OE SK-Hep1 cells (Figure 1, D and E, and Supplemental Figure 1B). Moreover, 2-phosphoglycerate, serine, and glycine levels were significantly increased in PCK1-OE cells, while TCA intermediates, including citrate and cis-aconitate, were reduced (Figure 1F and Supplemental Figure 1C). Further targeted LC-MS and quantitative analysis confirmed that serine, glycine, methionine, SAM, and SAH were reduced in PKO cells (Figure 1G) but accumulated in PCK1-OE cells (Figure 1H). SAM is a major methyl donor for all methylation reactions, including histone, RNA, and DNA methylation (6, 27). However, PCK1 had no significant effects on m6A abundance or 5mc or 5hmc modifications (Supplemental Figure 1, D–I), suggesting that the distinct methylation modification is associated with differential susceptibility to SAM levels and coregulation of multiple methyltransferases and demethylases (11). Therefore, we focused on histone methylation in our study. Taken together, these observations indicate that PCK1 catalyzes TCA intermediates into SSP, maintaining a relatively high level of SAM and H3K9me3 modification in hepatoma cells.

### PCK1 enhances H3K9me3 modification by SAM via the SSP and SUV39H1.

**Figure 2:**
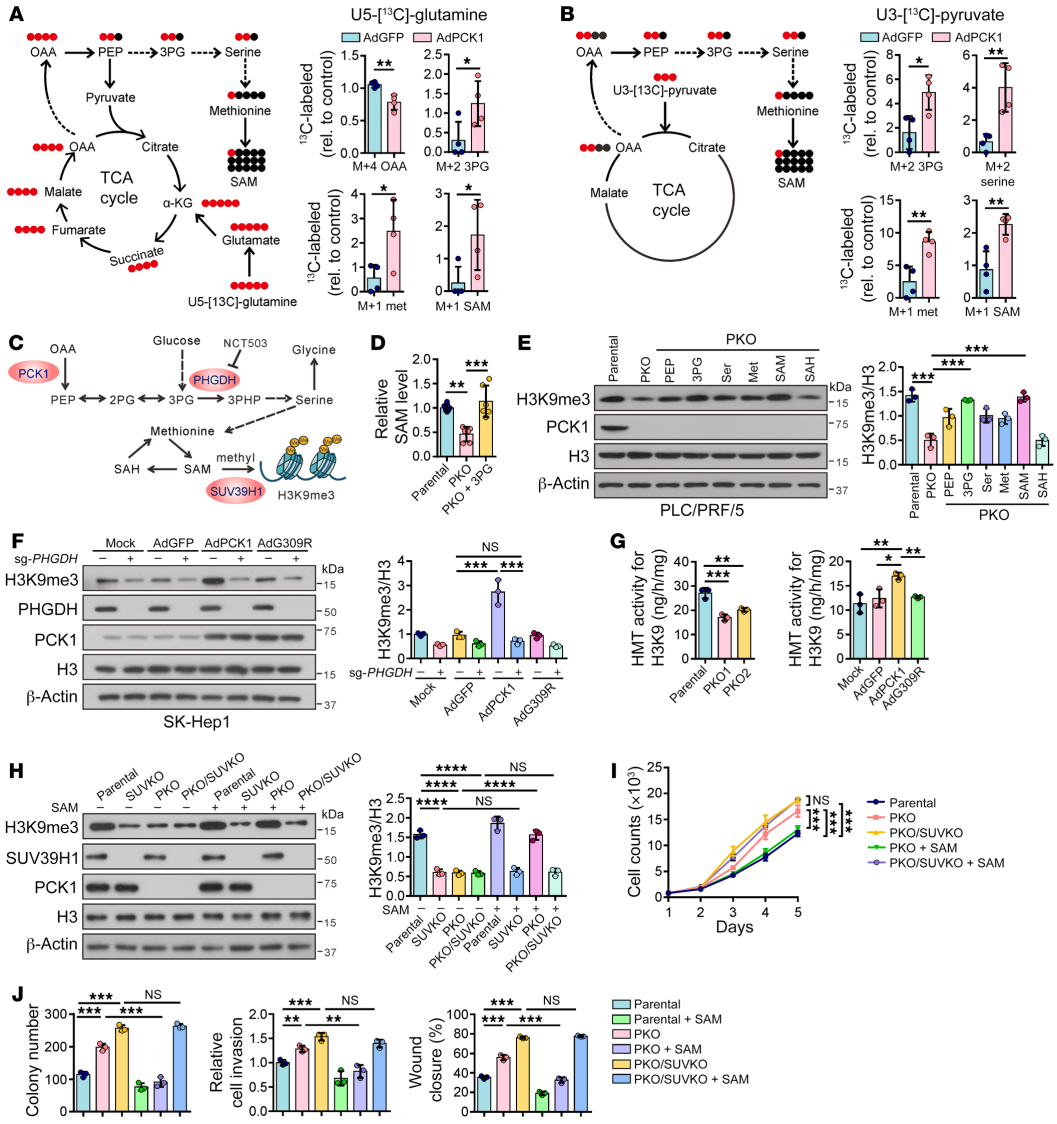
PCK1 enhances H3K9me3 modification by SAM via the SSP and SUV39H1. (**A**) Schematic diagram for the conversion of U-[^13^C]-glutamine into various metabolites and LC-MS profiles of M+4 OAA, M+2 3PG, M+1 methionine, M+1 SAM, respectively, after PCK1-OE cells were incubated with U-[^13^C]-glutamine for 24 hours (*n* = 4 biologically independent samples). (**B**) Schematic diagram for the conversion of U-[^13^C]-pyruvate into various metabolites and LC-MS profiles of M+2 3PG, M+2 serine, M+1 methionine, M+1 SAM, respectively, after PCK1-OE cells were incubated with U-[^13^C]-pyruvate for 24 hours (*n* = 4 biologically independent samples). (**C**) Schematic overview showing that PCK1-induced H3K9me3 modification depends on SAM accumulation derived from SSP. (**D**) Intracellular SAM in PKO cells in the presence or absence of 3PG (*n* = 6 biologically independent samples). (**E**) PKO cells were treated with PEP (0.5 mM), 3PG (0.75 mM), serine (400 μM), methionine (100 μM), SAM (50 μM), and SAH (50 μM), respectively, for 24 hours. Immunoblots for H3K9me3 were repeated 3 times independently with similar results. Data from 1 representative experiment are shown. Densitometric analysis of H3K9me3 was performed, normalized to histone H3. (**F**) Immunoblots for H3K9me3 modification in SK-Hep1 cells. Data from 1 representative experiment are shown (*n* = 3). (**G**) Histone methyltransferase (HMT) activities for H3K9 in PKO cells (left) and PCK1-OE cells (right) (*n* = 3 technical replicates). (**H**–**J**) PKO cells or PCK1/SUV39H1 double-KO cells (PKO/SUVKO cells) were treated with or without SAM for 24 hours, and (**H**) Western blot (*n* = 3), (**I**) cell growth curves (*n* = 3 technical replicates), and (**J**) colony formation assays, Transwell assays, and wound scratch assays (*n* = 3 biologically independent samples), are shown. Data are shown as the mean ± SEM. Statistical analysis was performed using 2-tailed unpaired Student’s *t* test (**A** and **B**), 1-way ANOVA with Tukey’s test (**D**–**H** and **J**), or 2-way ANOVA with Bonferroni’s test (**I**). *P < 0.05, **P < 0.01, ***P < 0.001, ****P < 0.0001.

To further elucidate whether PCK1 drives TCA intermediates into SSP flux that fuels SAM generation, we used the LC-MS–based ^13^C carbon–tracing method to characterize the dynamic metabolic activities. The results from uniformly labeled U-[^13^C]-glutamine tracing indicated that PCK1 mediated the conversion of TCA intermediates, including M+4 malate, M+4 fumarate, and M+4 OAA, while significantly increasing ^13^C carbon label incorporation into M+2 3-phosphoglycerate (3PG), M+1 methionine, and M+1 SAM (Figure 2A and Supplemental Figure 2A). Uniformly labeled U-[^13^C]-pyruvate tracing also indicated that PCK1 modulates the increase of M+2 3PG, M+2 serine, M+1 methionine, and M+1 SAM via diverting the TCA intermediates into the SSP (Figure 2B and Supplemental Figure 2B). We next used [^13^C_5_]-methionine labeling to track SAM production through the methionine cycle. However, there was no significant difference in M+5 SAM between PCK1-OE cells and the control group (Supplemental Figure 2C). In summary, these results suggest that PCK1 promotes the SSP flux for an increase in SAM synthesis. Notably, the addition of 3PG was sufficient to fully restore SAM levels in PKO cells, indicating that SAM accumulation was mainly derived from the SSP (Figure 2, C and D). We then directly added SAM and related metabolites, including PEP, 3PG, serine, methionine, or SAH, to PKO cells to confirm that SSP-derived SAM accumulation and concomitant changes in H3K9me3 modification are dependent on PCK1 (Figure 2, C and E). Exogenous 3PG and SAM treatment led to a dramatic increase in H3K9me3 levels (Figure 2E) and diminished PKO cell growth and migration (Supplemental Figure 2, D and E). Gluconeogenesis-derived 3PG, which ultimately enters the SSP, is catalyzed by phosphoglycerate dehydrogenase (PHGDH), the first rate-limiting enzyme of SSP (Figure 2C). PCK1 increased H3K9me3 levels, while targeting PHGDH by sg*PHGDH* or inhibitor NCT503 after AdPCK1 infection failed to enhance H3K9me3 levels (Figure 2F and Supplemental Figure 2F). Furthermore, both sg*PHGDH* and NCT503 partially rescued the proliferation and migration of PCK1-OE SK-Hep1 cells (Supplemental Figure 2, G and H). Together, these data highlight the role of PCK1 in driving TCA intermediate overflow into the SSP, thereby supporting SAM generation to induce H3K9me3 modifications and an antiproliferative phenotype.

**Figure 3:**
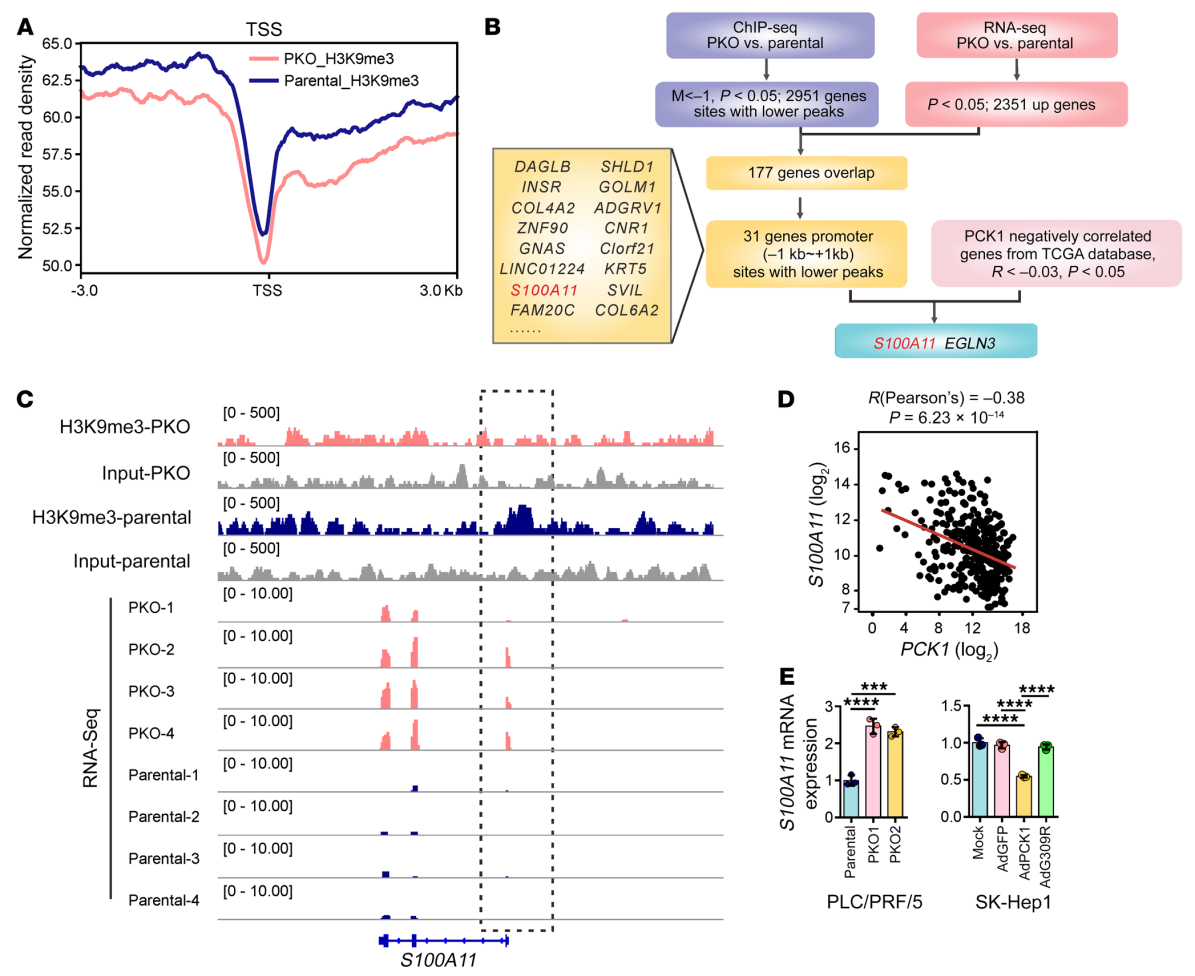
PCK1 suppresses *S100A11* transcription by increasing H3K9me3 occupancy. (**A**) Distribution of H3K9me3 ChIP-Seq signals in PKO cells around the transcription start site (TSS). (**B**) Bioinformatics analysis filtered *S100A11* as a downstream target of H3K9me3. up, upregulated. (**C**) Genome browser tracks of H3K9me3 occupancy and RNA-Seq (*n* = 4 biologically independent samples) at the *S100A11* gene locus. (**D**) Gene expression correlation between *S100A11* and *PCK1* (*r* = –0.38, *P* = 6.23 × 10^−14^) from the TCGA database. (**E**) qPCR analysis of *S100A11* expression in PKO cells (left) and PCK1-OE cells (right) (*n* = 3 technical replicates). Data are shown as the mean ± SEM. Statistical analysis was performed using 1-way ANOVA with Tukey’s test (**E**). ***P < 0.001, ****P < 0.0001.

In addition to the availability of methyl donors, histone methylation is dynamically regulated by various methyltransferases. Previous studies have found that SUV39H1, an important H3K9me3 methyltransferase with a higher Km value compared with other methyltransferases, could be responsible for the susceptibility of H3K9me3 to SAM restriction (10, 28). We observed that the activity of H3K9 methyltransferase was reduced in PKO cells and enhanced in PCK1-OE cells (Figure 2G). To determine the contribution of SUV39H1 to PCK1-dependent H3K9me3, SUV39H1 expression was depleted using sgRNA (SUV-KO cells). Notably, exogenous addition of SAM (Figure 2H) or 3PG (Supplemental Figure 2I) dramatically restored H3K9me3 levels in PKO cells, whereas no major difference was observed in PKO/SUV-KO cells. Supplying SAM (Figure 2, I and J) or 3PG (Supplemental Figure 2, J and K) to PKO but not PKO/SUV-KO cells reduced the growth and migration abilities of HCC cells, indicating that SUV39H1 is the primary methyltransferase involved in mediating H3K9me3 modification in these hepatoma cells. Overall, these data reveal that PCK1 promotes H3K9me3 modification and the antitumorigenesis phenotype, which depends on the enhanced activity of H3K9 methyltransferase SUV39H1 and the supply of methyl donor SAM via the SSP.

### PCK1 suppresses S100A11 by increasing SAM-dependent H3K9me3 occupancy.

**Figure 4:**
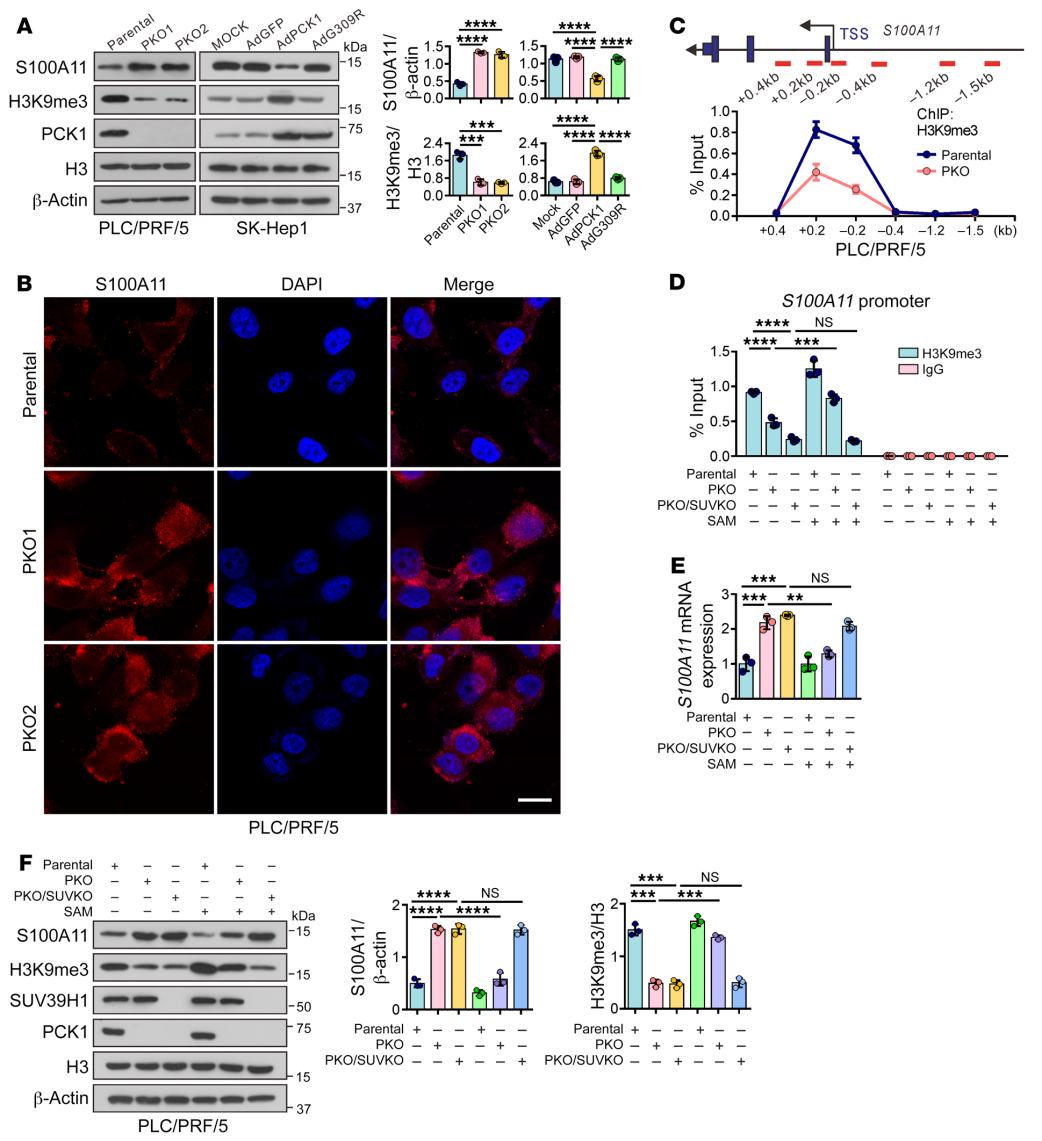
PCK1 suppresses S100A11 by increasing SAM-dependent H3K9me3 occupancy. (**A**) Representative blots and densitometries of H3K9me3 and S100A11 in PKO cells and PCK1-OE cells; histone H3 and β-actin were used as loading controls (*n* = 3). (**B**) Immunofluorescent images for S100A11 in PKO cells. (**C**) ChIP-qPCR showing the enrichment of H3K9me3 in different promoter regions of *S100A11* in PKO cells (*n* = 3 technical replicates). (**D**–**F**) PKO and PKO/SUVKO cells were supplemented with or without SAM for 24 hours, followed by (**D**) ChIP-qPCR analysis (*n* = 3 technical replicates), (**E**) qPCR assays (*n* = 3 technical replicates), and (**F**) Western blot detection (*n* = 3 times). Scale bars: 15 μm (**B**). Data are shown as the mean ± SEM. Statistical analysis was performed using 1-way ANOVA with Tukey’s test (**A** and **D**–**F**). **P < 0.01, ***P < 0.001, ****P < 0.0001.

To determine the regulatory role of H3K9me3 in gene expression in hepatoma cells, we first performed ChIP followed by sequencing (ChIP-Seq) using anti-H3K9me3 antibodies in PLC/PRF/5 PKO and parental cells. ChIP-Seq data showed that H3K9me3 enrichment was largely reduced in PKO cells among the promoter regions (Figure 3A). As H3K9me3 is typically associated with transcriptional repression, we combined ChIP-Seq with RNA-Seq data and identified 31 target genes with low H3K9me3 occupancy at the promoter region (–1 kb to +1 kb) and high mRNA levels in PKO cells (Figure 3B). Among these candidate genes, *S100A11* (*r* = −0.38, *P* = 6.23 × 10^−14^), has the highest negative correlation with *PCK1* in The Cancer Genome Atlas (TCGA) database (Figure 3, B–D) and has been reported to act as an oncogene in several tumors (29–31). Recent studies have found that *S100A11* is associated with high-grade HCC and poor prognosis through its promotion of cancer cell proliferation and migration (32). Thus, we decided to focus on S100A11 to elucidate the mechanism underlying PCK1 deficiency–induced HCC. Notably, S100A11 was highly expressed in PKO cells at both the mRNA (Figure 3E and Supplemental Figure 3A) and protein levels (Figure 4, A and B, and Supplemental Figure 3B) but was reduced in PCK1-OE cells at both the mRNA (Figure 3E and Supplemental Figure 3A) and protein levels (Figure 4A and Supplemental Figure 3, B and C). ChIP-qPCR assays also showed that H3K9me3 was enriched in the *S100A11* promoter region, and this enrichment was reduced by PCK1 KO (Figure 4C and Supplemental Figure 3D), supporting the hypothesis that PCK1 represses *S100A11* expression through H3K9me3 modification.

Furthermore, H3K9me3 levels increased at the *S100A11* promoter upon the addition of SAM or 3PG (Supplemental Figure 3, E and F). As expected, SAM or 3PG remarkably downregulated both mRNA (Supplemental Figure 3, G and H) and protein levels (Supplemental Figure 3, I and J) of S100A11. Conversely, the PHGDH inhibitor NCT503 blocked 3PG transfer into the SSP and SAM generation, which led to a marked reduction in the enrichment of H3K9me3 on *S100A11* in PCK1-OE cells (Supplemental Figure 3K). Accordingly, *S100A11* mRNA and protein levels were restored by NCT503 treatment (Supplemental Figure 3, L and M). Moreover, SUV39H1 is the primary methyltransferase involved in PCK1-dependent H3K9me3 modification, and we found that PKO cells treated with SAM, but not PKO/SUV-KO cells, restored H3K9me3 enrichment on *S100A11* promoter (Figure 4D). In contrast, supplying SAM to PKO cells attenuated both the mRNA and protein levels of S100A11, while no obvious change was found in PKO/SUV-KO cells in the presence of SAM (Figure 4, E and F). Collectively, these results suggest that PCK1 suppresses the transcription of oncogene *S100A11* in an H3K9me3-dependent manner.

### PCK1 deficiency induces HCC cell proliferation, migration, and tumorigenesis via S100A11.

**Figure 5:**
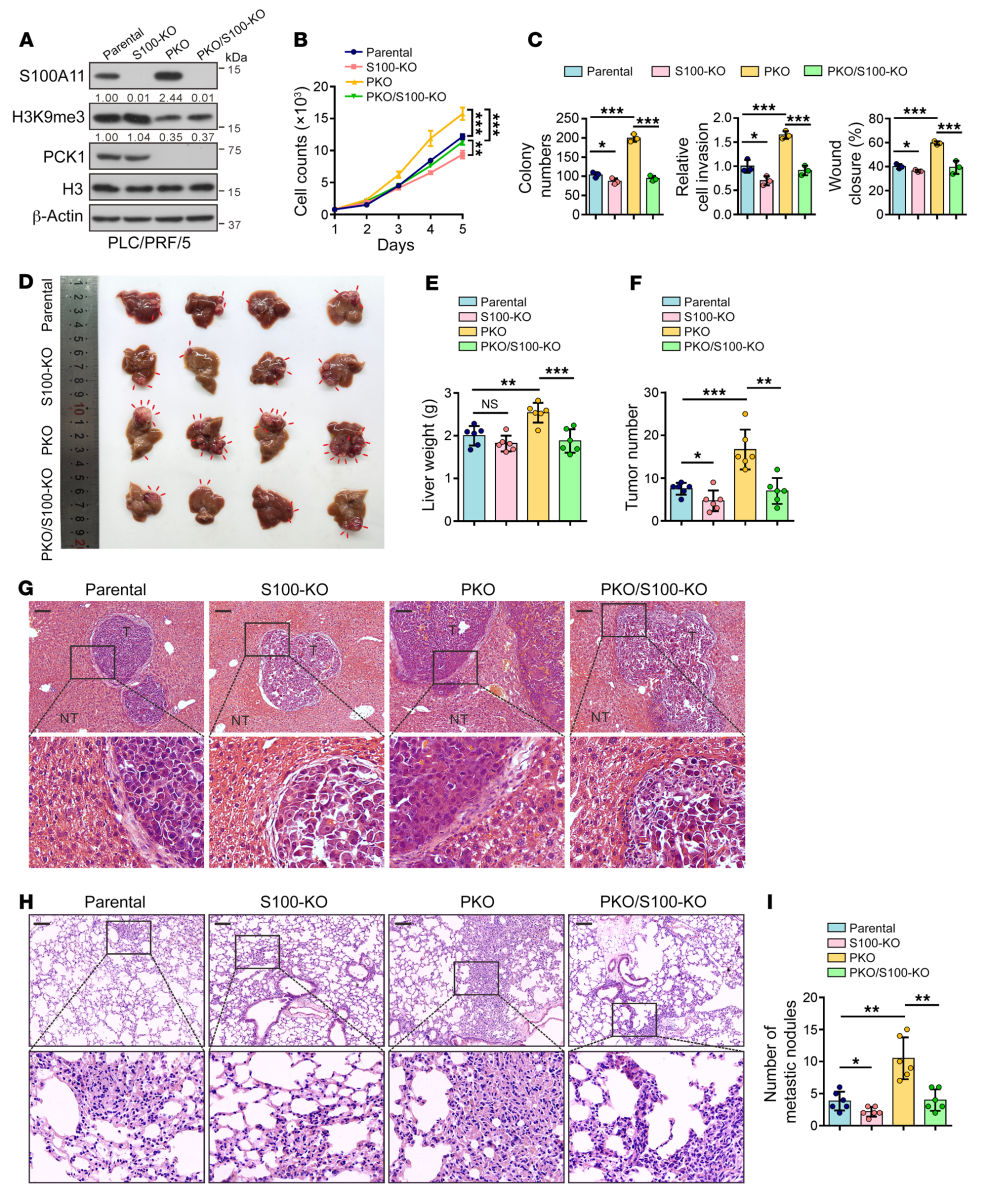
PCK1 deficiency induces HCC cell proliferation, migration, and tumorigenesis via S100A11. (**A**) Western blot showing the protein expression from PCK1/S100A11 double-KO PLC/PRF/5 cells (PKO/S100-KO cells). Numbers that appear below the blots represent the relative densities (measured using ImageJ software) of S100A11 protein bands normalized to β-actin, or the relative densities of H3K9me3 modification normalized to histone H3. (**B**) Cell proliferation (*n* = 3 technical replicates) and (**C**) colony formation assays, Transwell assays, and wound-healing assays (*n* = 3 biologically independent samples) in PKO/S100-KO cells. (**D**) Gross images, (**E**) liver weight, (**F**) tumor number, and (**G**) H&E staining in the orthotopic HCC model, as indicated (*n* = 6 mice per group). (**H**) H&E staining analysis and (**I**) quantification of metastatic nodules in the lung metastasis model (*n* = 6 mice per group). Scale bars: 100 μm (**G** and **H**). Data are shown as the mean ± SEM. Statistical analysis was performed using 1-way ANOVA with Tukey’s test (**C**, **E**, **F**, and **I**) and 2-way ANOVA with Bonferroni’s test (**B**). *P < 0.05, **P < 0.01, ***P < 0.001.

Given the role of PCK1 in suppressing hepatocarcinogenesis, proliferation, and epithelial-mesenchymal transition, we investigated whether PCK1 depletion–induced phenotypes were due to the enhancement of oncogene S100A11. Next, we examined the role of S100A11 in PKO cells using sgRNA targeting the *S100A11* gene (sg*S100A11*) (Figure 5A). As expected, S100A11 depletion reduced the proliferation (Figure 5B) and colony formation (Figure 5C) of PKO cells. Moreover, Transwell and wound healing (Figure 5C) assays showed that silencing S100A11 markedly impaired the invasive and migratory capacities of PKO cells. To further investigate the tumorigenic function of S100A11 in vivo, hepatoma cells infected with lentiviral sg*S100A11* or lentiviral sgCtrl were transplanted into the livers of nude mice to establish orthotopically implanted HCC models (Supplemental Figure 4A). We showed that S100A11 KO significantly reduced tumor burden in PKO hepatoma cells (Figure 5, D–G), indicating that S100A11 depletion effectively inhibited tumor growth in vivo. Moreover, in tail-vein models of lung metastasis in nude mice, fewer metastatic foci were observed in tissue sections of the lungs in PKO cells infected with lentiviral sg*S100A11* (Figure 5, H and I), illustrating that the lung metastatic potential of PCK1-deficient hepatoma cells could be facilitated by S100A11.

We further ascertained whether PCK1-induced tumor suppression could be rescued by S100A11. As expected, S100A11 overexpression restored the proliferation and migration of PCK1-OE SK-Hep1 cells (Supplemental Figure 4, B–D). Likewise, the impaired tumorigenesis potential triggered by PCK1 in vivo could be restored by S100A11 overexpression (Supplemental Figure 4, E–I). Taken together, these findings demonstrate that S100A11 functions as a key oncogenic driver in PCK1 deficiency–induced HCC cell proliferation, migration, and tumorigenesis.

### Loss of PCK1 activates PI3K/AKT signaling through S100A11.

**Figure 6:**
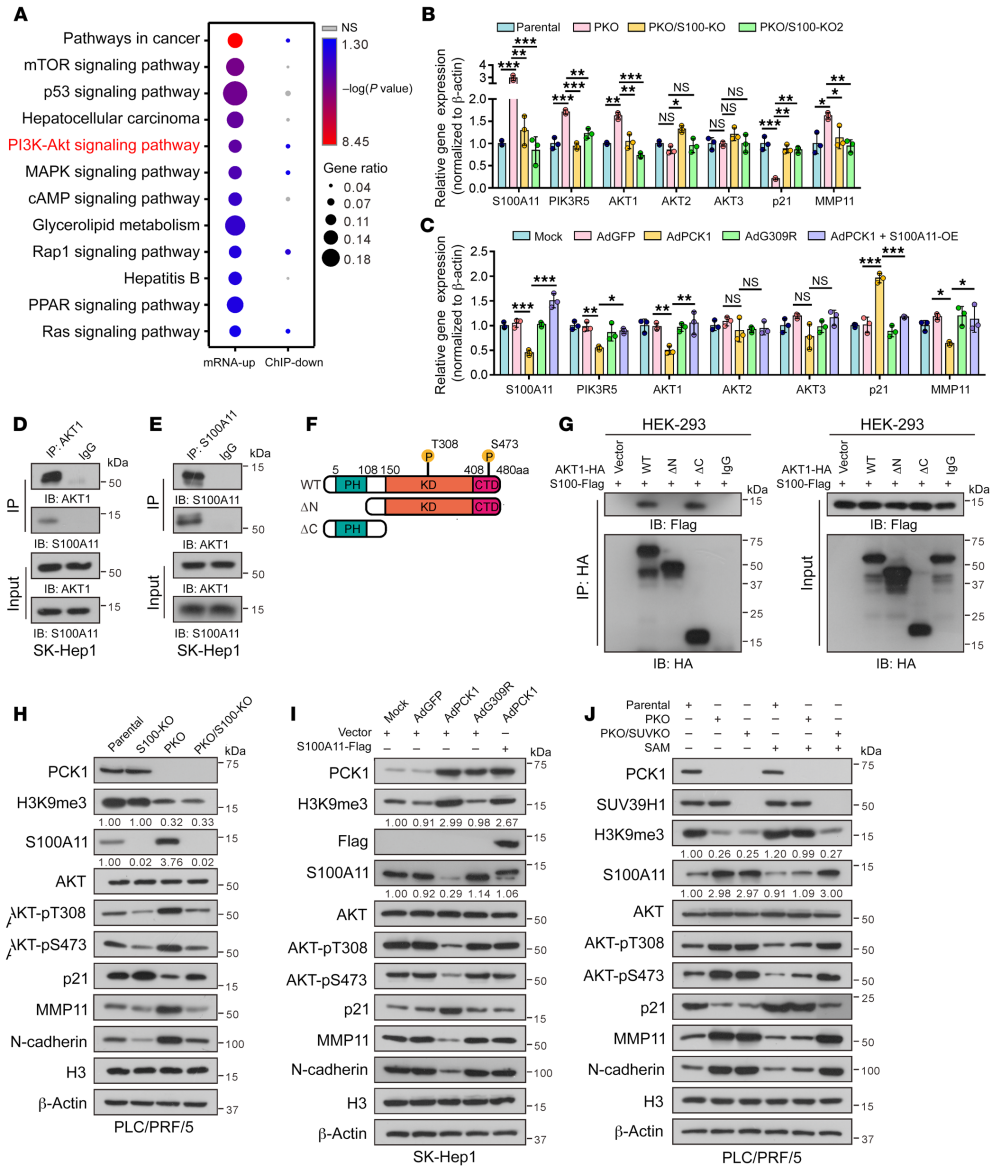
Loss of PCK1 activates PI3K/AKT signaling through S100A11. (**A**) RNA-Seq (*n* = 4 biologically independent samples) combined with ChIP-Seq analysis filtered PI3K/AKT signaling. (**B** and **C**) The mRNA levels of PI3K/AKT signaling genes and associated markers were assayed by qPCR (**B**) in PKO/S100-KO cells and (**C**) PCK1-OE cells transfected with S100A11-overexpressing plasmid (*n* = 3 technical replicates). (**D** and **E**) Immunoprecipitated proteins, with the indicated antibodies, subjected to immunoblotting analysis. (**F**) Schematic representation of the AKT1 constructs. WT AKT1 contains 3 domains, the pleckstrin homology (PH) domain, kinase domain (KD), and C-terminal domain (CTD). Truncation mutants of AKT1, comprising amino acids 108–480 or 1–151, were designated as ΔN and ΔC, respectively. (**G**) Interactions between S100A11 and full-length protein (aa 1–480), the ΔN truncation mutant (aa 108–480), or the ΔC truncation mutant (aa 1–151) in HEK-293 cells were determined by Co-IP. (**H** and **I**) Immunoblots of samples from (**H**) PKO/S100-KO cells and (**I**) PCK1-OE cells transfected with S100A11-overexpressing plasmid. Numbers that appear below the blots represent the relative densities (measured using ImageJ software) of S100A11 protein bands normalized to β-actin, or the relative densities of H3K9me3 modification normalized to histone H3. (**J**) Western blots for PKO cells or PKO/SUV-KO cells treated with or without SAM for 24 hours. Data are shown as the mean ± SEM. Statistical analysis was performed using 1-way ANOVA with Tukey’s test (**B** and **C**) or log-rank test (**A**); *P < 0.05, **P < 0.01, ***P < 0.001.

To define the tumorigenic mechanism underlying PCK1 deletion–induced S100A11 upregulation, we combined ChIP-Seq with RNA-Seq data and analyzed the relevant pathways from the Kyoto Encyclopedia of Genes and Genomes and found that the PI3K/AKT pathway was the most significantly enriched (Figure 6A and Supplemental Figure 5, A and B). We validated that PCK1 KO upregulated the PI3K/AKT pathway, whereas PCK1/S100A11 double KO reversed this change (Figure 6B and Supplemental Figure 5, C and D). Conversely, PCK1 overexpression significantly inactivated the PI3K/AKT pathway, which was rescued by the reexpression of S100A11 (Figure 6C).

Previous observations have shown that S100A11 has 2 EF-hand motifs, which are composed of 2 α-helices and a Ca^2+^ binding loop (33). Ca^2+^ binding induces a conformational rearrangement that exposes a hydrophobic cleft, allowing the S100 protein to bind to its cellular targets and elicit a physiological response (34). Moreover, S100A11-induced AKT phosphorylation has been reported previously (35, 36). Thus, we considered whether S100A11 activates PI3K/AKT signaling by directly interacting with AKT1. To test this hypothesis, we validated the association of S100A11 with AKT1 by a coimmunoprecipitation (Co-IP) assay in SK-Hep1 cells (Figure 6, D and E) and HEK-293 cells (Supplemental Figure 5, E and F). Immunofluorescence analysis also indicated that S100A11 and AKT1 colocalized in the plasma membrane (Supplemental Figure 5G). We next examined the precise region(s) of S100A11-AKT1 association and found that it is likely mediated through the pleckstrin homology (PH) domain of AKT1 (Figure 6, F and G).

As expected, S100A11 KO caused a detectable reduction in AKT activation in PKO cells (Figure 6H). SAM (Supplemental Figure 5H) or 3PG supplementation (Supplemental Figure 5I) inhibited AKT pathway activation. In contrast, introducing S100A11 back in PCK1-OE cells led to AKT activation (Figure 6I). In addition, the PHGDH inhibitor NCT503 blocked the conversion of 3PG to SSP, which rescued the phosphorylation of AKT in PCK1-OE cells (Supplemental Figure 5J). Importantly, we found that supplying SAM to PKO cells remarkably attenuated the phosphorylation of AKT, while no obvious change was found in PKO/SUV-KO cells treated with SAM (Figure 6J). These results suggest that S100A11 governs hyperactivation of the PI3K/AKT signaling pathway in PCK1-KO cells.

### Reductive H3K9me3 modification at S100a11 promotes DEN/CCl_4_/PB-induced hepatocellular carcinogenesis in Pck1-KO mice.

**Figure 7:**
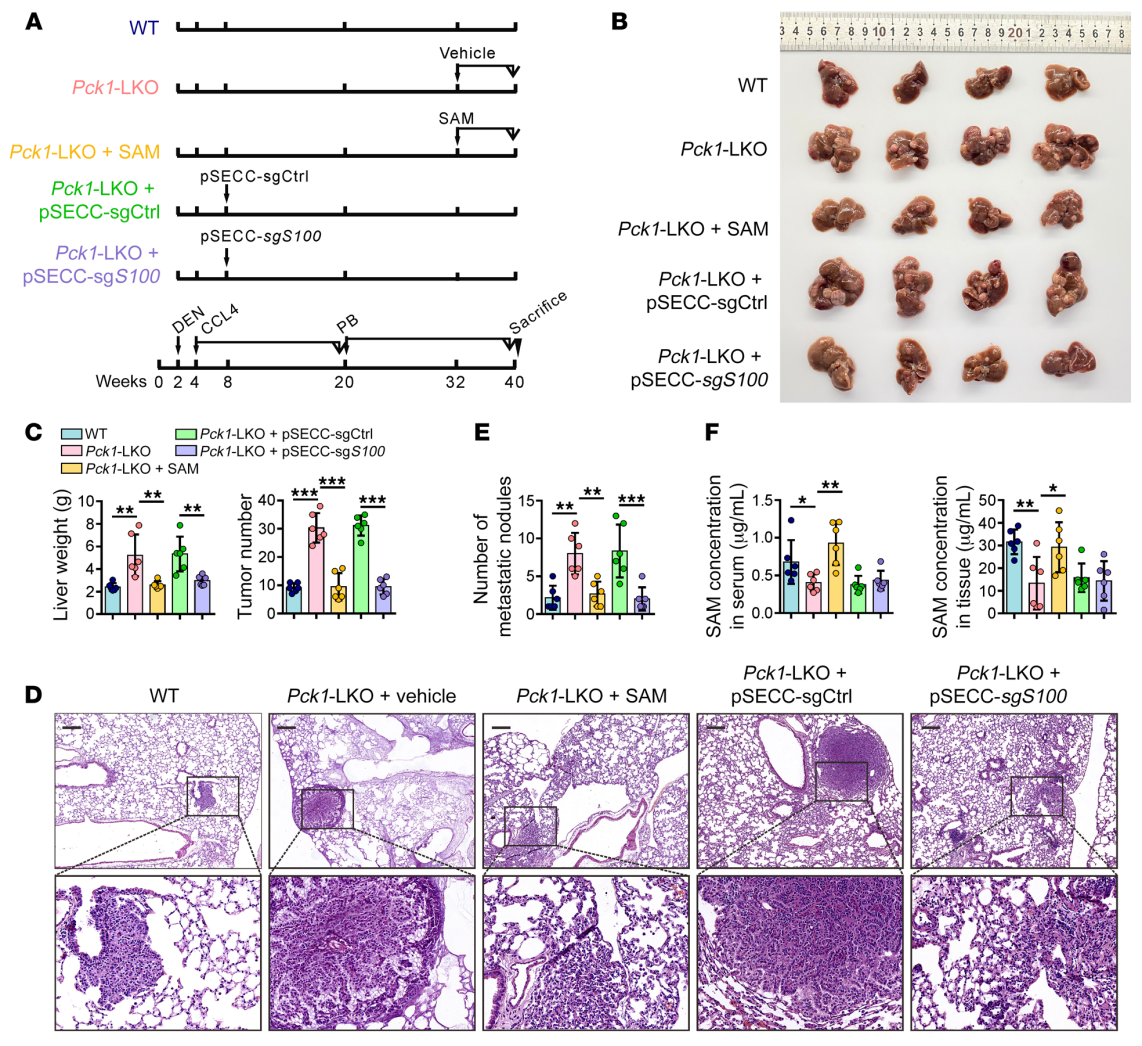
The progression of DEN/CCl4/PB-induced hepatocellular carcinogenesis in *Pck1*-KO mice is suppressed by SAM supplement or *S100a11* KO. (**A**) Scheme of the experimental procedure with *AlbCre^–/–^ Pck1^fl/fl^* (WT), *AlbCre^+/–^ Pck1^fl/fl^* mice (liver-specific KO [LKO]) (*n* = 6 mice per group). (**B**) Gross images and (**C**) quantification of weight and numbers of tumor nodules of livers from WT, *Pck1*-LKO treated with vehicle, *Pck1*-LKO treated with SAM, *Pck1*-LKO injected with pSECC-sgControl, and *Pck1*-LKO injected with pSECC-sg*S100a11* mice (*n* = 6 mice per group). (**D**) Representative images of H&E staining for lung tissues were provided, and (**E**) the metastatic foci were counted (*n* = 6 mice per group). (**F**) Ultra-performance liquid chromatography results for the SAM concentration in mouse serum samples and liver tissues (*n* = 6 mice per group). Scale bars: 200 μm (**D**). Data are shown as the mean ± SEM. Statistical analysis was performed using 1-way ANOVA with Tukey’s test (**C**, **E**, and **F**). *P < 0.05, **P < 0.01, ***P < 0.001.

**Figure 8:**
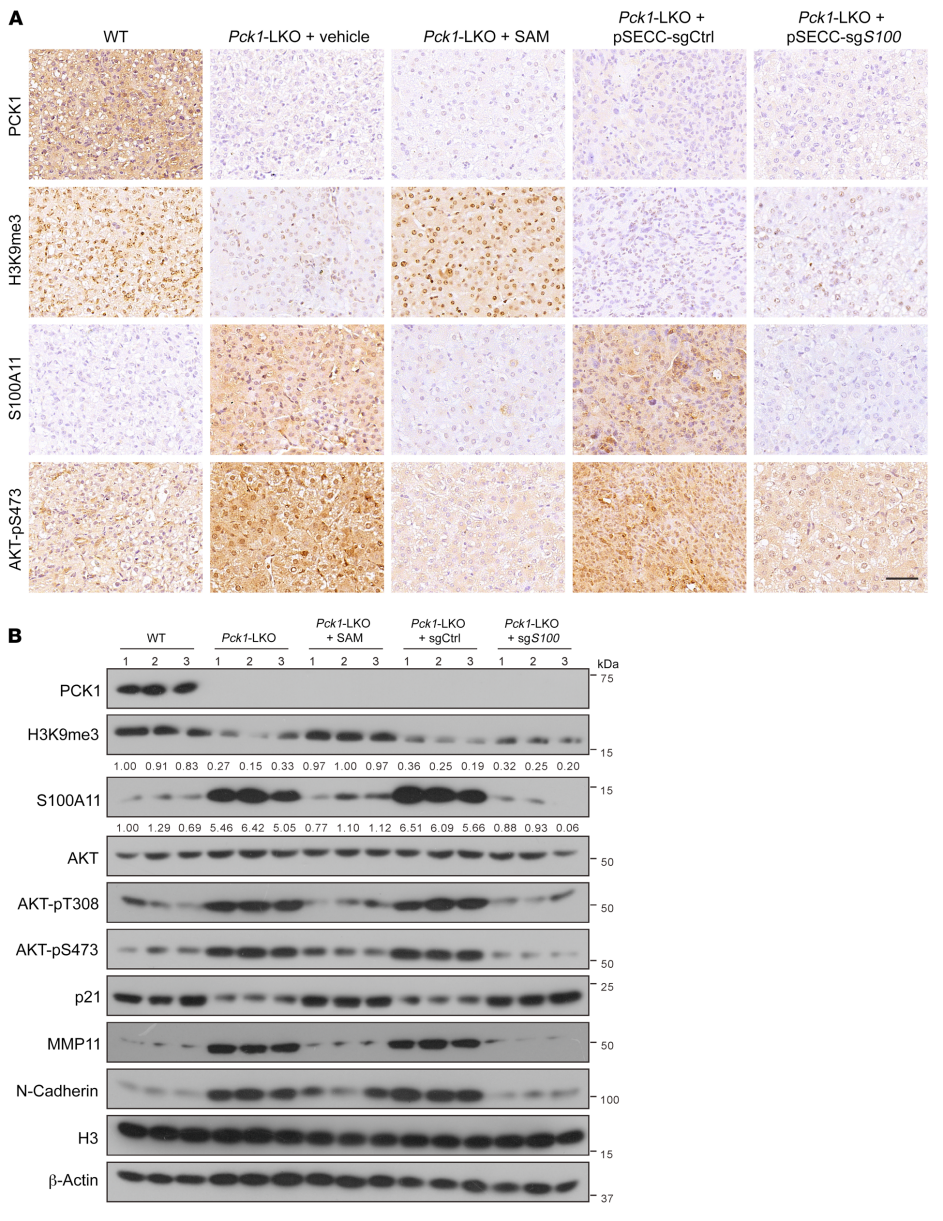
Reductive H3K9me3 modification at *S100a11* promotes DEN/CCl_4_/PB-induced hepatocellular carcinogenesis in *Pck1*-KO mice. (**A**) Indicated proteins or modifications were detected by immunohistochemical assays and (**B**) Western blot detection. Numbers that appear below the blots represent the relative densities (measured using ImageJ software) of S100A11 protein bands normalized to β-actin, or the relative densities of H3K9me3 modification normalized to histone H3 Scale bars: 50 μm (**A**).

To further confirm that PCK1 exerted antitumor effects through S100A11 suppression via upregulation of H3K9me3 modification in vivo, we developed a DEN/CCl_4_/PB-induced mouse model of liver cancer (Figure 7A and Supplemental Figure 6A) (37). In the LKO + SAM group, 8-month-old LKO mice were treated with SAM (100 mg/kg/d, 4 days per week) for 8 weeks (Figure 7A). In the pSECC-sg*S100a11*–treated group, LKO mice were intravenously infected with pSECC-sg*S100a11* lentiviruses at 8 weeks (Figure 7A and Supplemental Figure 6, B and C). DEN/CCl_4_/PB administration led to increased liver tumorigenesis and lung metastasis in the LKO group compared with those in the WT group (Figure 7, B–E, and Supplemental Figure 6D). However, LKO mice treated with SAM or depleted *S100a11* showed a significant reduction in tumor volume and number as well as lung metastatic nodules compared with those of untreated LKO mice (Figure 7, B–E). SAM levels were lower, whereas alanine aminotransferase and aspartate aminotransferase levels were significantly higher in the sera of untreated LKO mice (Figure 7F and Supplemental Figure 6E). Importantly, SAM supplementation resulted in a marked upregulation of H3K9me3 and p21 protein levels but a reduction in the levels of S100A11, p-AKT, and MMP11 proteins (Figure 8 and Supplemental Figure 6F). Collectively, these data demonstrated that PCK1 depletion induces S100A11 expression and activates the PI3K/AKT pathway to increase susceptibility to DEN/CCl_4_/PB-induced HCC progression.

### Correlation among PCK1, H3K9me3, and S100A11 expression in HCC specimens.

**Figure 9:**
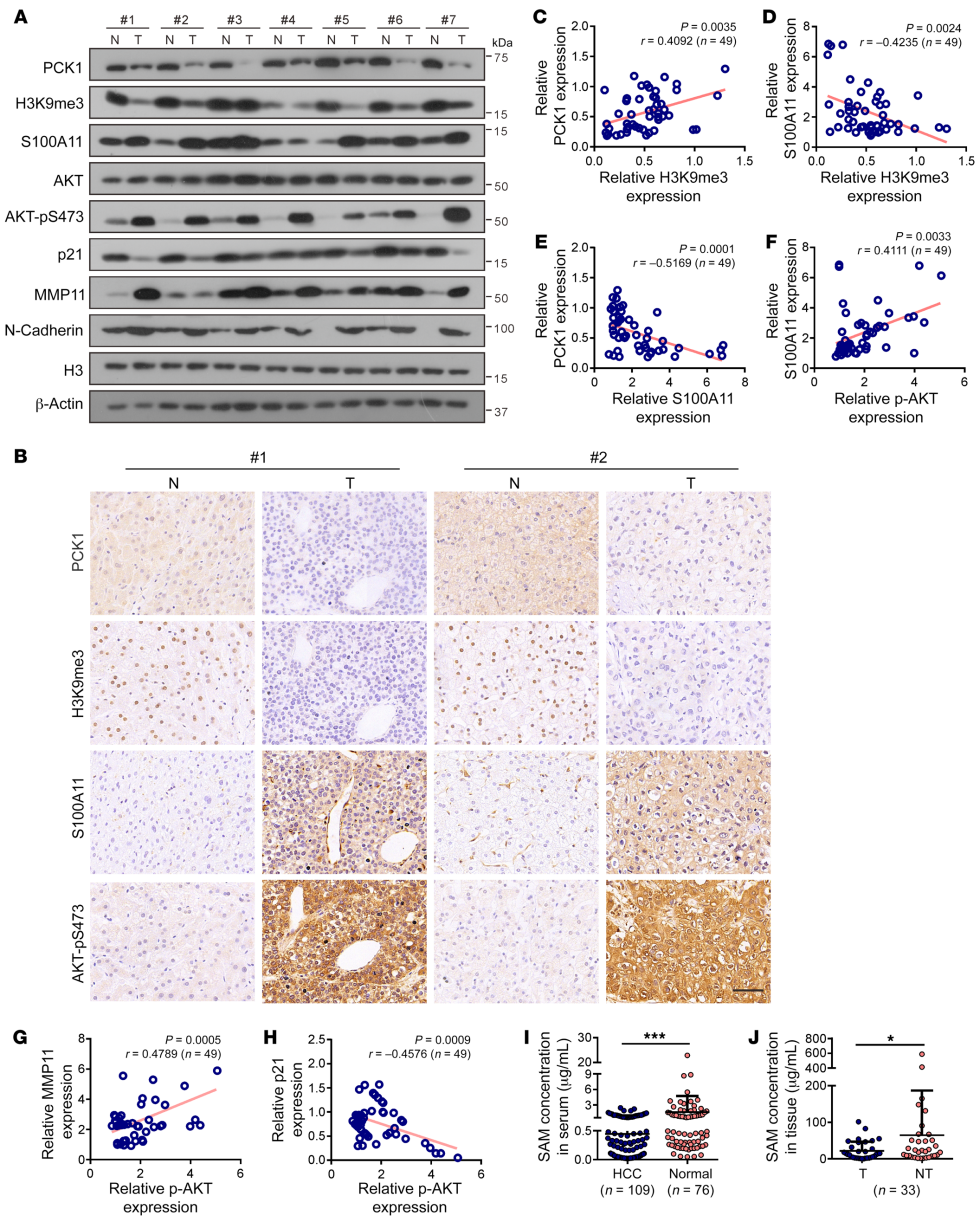
Correlation among PCK1, H3K9me3, and S100A11 expression in HCC specimens. (**A** and **B**) Indicated proteins or modification levels in representative human HCC specimens and surrounding nontumorous tissues were measured by (**A**) Western blot in 7 HCC patients and (**B**) IHC staining in 2 HCC patients (serial sections). N, surrounding nontumorous tissues; T, HCC specimens. (**C**–**H**) Correlation analysis between indicated protein expression in tumor tissues from 49 patients with HCC (see also Supplemental Figure 7, A–F). Correlation analysis between (**C**) H3K9me3 and PCK1, (**D**) H3K9me3 and S100A11, (**E**) S100A11 and PCK1, (**F**) S100A11 and AKT-pS473, (**G**) AKT-pS473 and MMP11, and (**H**) AKT-pS473 and p21. (**I** and **J**) ultra-performance liquid chromatography results for the SAM concentration in (**I**) serum samples (HCC, *n* = 109; normal *n* = 76) and (**J**) HCC tissues and paired adjacent liver tissues (*n* = 33). Scale bars: 50 μm (**B**). Data are shown as the mean ± SEM. Statistical analysis was performed using 2-tailed unpaired Student’s *t* test (**I**), 2-tailed paired Student’s t test (**J**), or Pearson’s correlation coefficient (**C**–**H**). *P < 0.05, ***P < 0.001.

We further examined the expression levels of PCK1, H3K9me3, and S100A11 in 49 pairs of HCC samples and adjacent normal tissues. The results revealed that PCK1, H3K9me3, and p21 expression in HCC tissues was substantially lower than that in normal tissues (Figure 9, A and B, and Supplemental Figure 7, A–F). Conversely, S100A11, p-AKT, MMP11, and N-cadherin expression was higher in tumor tissues than in adjacent normal tissues (Figure 9, A and B, and Supplemental Figure 7, A–F). Moreover, correlation analysis revealed that PCK1 protein abundance was positively correlated with H3K9me3 in HCC tumor samples (Figure 9C, *r* = 0.4092, *P* = 0.0035), while negative correlations were observed between S100A11 and H3K9me3 (Figure 9D, *r* = –0.4235, *P* = 0.0024) and PCK1 (Figure 9E, *r* = –0.5169, *P* = 0.0001). Consistent with our in vitro data, we observed positive correlations between S100A11 and p-AKT expression (Figure 9F, *r* = 0.4111, *P* = 0.0033) and p-AKT and MMP11 expression (Figure 9G, *r* = 0.4789, *P* = 0.0005), while there was a negative correlation between p-AKT and p21 expression (Figure 9H, *r* = –0.4576, *P* = 0.0009). To further validate our observations, we quantified SAM levels in 109 serum samples from patients with HCC and 76 healthy controls (Figure 9I) and SAM levels in 33 pairs of HCC tissues and adjacent liver tissues (Figure 9J). The results revealed that the average SAM levels were much lower in both the HCC sera (Figure 9I, *P* = 0.0003) and tissues (Figure 9J, *P* = 0.0398). Next, we analyzed TCGA liver cancer cohorts and found that *S100A11* mRNA expression was higher in HCC (Supplemental Figure 7G) and was associated with poor HCC progression (Supplemental Figure 7H). Moreover, survival analysis showed that patients with lower levels of *PCK1* and higher levels of *S100A11* had poorer overall survival (Supplemental Figure 7I). Collectively, these results support the finding that PCK1 deficiency alleviates SAM biosynthesis and H3K9me3 modification in S100A11, thereby promoting aberrant PI3K/AKT activation and tumor progression in human primary HCC.

## Discussion

Accumulating evidence indicates that dysregulated metabolism is closely linked with epigenetic remodeling, enabling tumor progression (38, 39). However, how fluctuating levels of metabolites specifically reshape epigenetic alterations and gene expression remains unclear. Our results strongly suggest that PCK1-dependent generation of SAM is important for HCC suppression by enhancing H3K9me3 modification of the *S100A11* promoter and downregulating the PI3K/AKT signaling pathway. Previous studies have demonstrated that elevated PCK1 expression is responsible for decreasing cellular ATP levels that activate p-AMPK (19), while deletion of PCK1 leads to accumulated ribose-5-phosphate (R5P) that protects cells against oxidative damage (18) and enhanced UDP-GlcNAc biosynthesis that upregulates O-GlcNAcylation (21). Moreover, a recent study on T cell memory homeostasis found that PCK1 boosts the biosynthesis of G6P and R5P along the gluconeogenic pathway, which ensures the quenching of ROS to facilitate the survival of CD8^+^ Tm cells (40). Nevertheless, our study revealed that PCK1 drives TCA intermediates into the SSP, maintaining a relatively high level of SAM. Increased levels of SAM enhance H3K9me3 histone methylation via the methyltransferase SUV39H1, thereby suppressing the transcription and expression of the oncogene *S100A11*. The loss of PCK1 results in the enhancement of S100A11, which recruits AKT1 to facilitate the activation of the PI3K/AKT signaling pathway, thereby promoting tumor progression. We believe that these findings elaborate a potential regulatory mechanism of PCK1 in HCC and provide insights on how fluctuating SAM levels modulate the expression of specific genes. These results highlight the association between metabolic reprogramming and epigenetic modifications for more effective therapeutic prospects in HCC.

SAM is generated during 1-carbon metabolism (e.g., serine, glycine, threonine, and methionine metabolism) and the metabolism of other intermediate metabolites derived from the TCA cycle (38, 41). As a universal methyl donor, SAM is required by all methyltransferases to promote histone methylation. Our study underlines the modulatory effect of PCK1 on SAM levels, which depends on channeling the gluconeogenesis intermediate 3PG into the SSP. Both PCK1 and PCK2 contribute to the enrichment of 3PG (18, 42), a succeeding intermediate after PEP in the gluconeogenic pathway, which could channel into SSP to facilitate SAM biosynthesis (18, 43). H3K9me3 is one of the most dynamic histone methylation markers and is highly dependent on relatively high SAM concentrations. Recent studies have revealed that dysregulation of H3K9me3 plays an important role in both human and mouse HCC (25, 26, 44, 45). A positive correlation between PCK1 expression and H3K9 methyltransferase activity was observed in the present study. Thus, we suggest that SUV39H1, which catalyzes histone H3K9me3 and has a higher Km value compared with other methyltransferases, is responsible for the susceptibility of H3K9me3 to SAM availability in hepatoma cells.

Recent studies have shown that SAM supplementation can inhibit cell proliferation and invasion by causing hypermethylation-mediated inactivation of prometastatic genes (46–48). However, other studies show that SAM treatment does not result in a significant global DNA methylation change while the expression of genes involved in tumor growth was reduced (49, 50). Here, we found that the gluconeogenic enzyme PCK1 upregulates SAM levels in vitro and in vivo. Consistent with a previous study (51), we detected lower levels of SAM in HCC tissues than in paired primary tumors and lower levels of SAM in HCC sera than in healthy controls. Intriguingly, SAM administration effectively alleviated HCC progression and metastasis in LKO mice. PCK1 promotes H3K9me3 modification and the antitumorigenesis phenotype, which may be due to greater sensitivity to the multiple upstream metabolic flux and downstream SAM availability required for the reciprocal regulation between metabolic rewiring and epigenetic remodeling.

SAM is synthesized from methionine catalyzed by methionine adenosyltransferases (MATs) (52). These enzymes are encoded by MAT1A (encode isozymes MATI/III), which is mainly expressed in the normal liver, and MAT2A (encode MATII), which is expressed in all extrahepatic tissues at a relatively low level (13). High expression of MAT2A in extrahepatic tissue increases the production of SAM, in which case increased SAM regulates gene expression by epigenetic mechanisms as well as enhancing cell proliferation and migration in various tumors, such as colon cancer, breast cancer, and non–small cell lung cancer (53–56). However, HCC is characterized by the switch of decreased MAT1A to increased MAT2A, which contributes to reduced SAM concentration and subsequently favors the proliferative signaling (52). Thus, the complicated role of SAM may be involved in the metabolic vulnerabilities of MAT2A enzyme or SAM levels in HCC and other cancers.

Additionally, we identified that high levels of SAM increased H3K9me3, which is responsible for repressing expression of *S100A11* and downregulation of the PI3K/AKT pathway. In mouse models, KO of S100A11 restricted the development of HCC in LKO mice. S100A11 is considered an oncogenic factor that contributes to cell proliferation and growth via diverse signaling pathways (33) and mediates metastatic tissue invasion by facilitating membrane translocation or plasma membrane repair (31, 57). Its conformational rearrangement and subsequent exposure of hydrophobic residues allow S100A11 to participate in target protein binding (57, 58). The AKT kinase family includes 3 homologous isoforms: AKT1, AKT2, and AKT3. AKT1 is ubiquitously expressed, whereas AKT2 and AKT3 are the major isoforms expressed in insulin-responsive tissues and the brain, respectively (59). Without C-terminal phosphorylation, AKT1 exists in an inactive conformation with autoinhibition involving a PH domain–kinase domain interaction (60). In the present study, we demonstrated that S100A11 directly interacts with AKT1 to activate PI3K/AKT signaling, initiating proliferation and migration in HCC. Further studies are required to assess whether the subcellular location and Ca^2+^-mediated conformational rearrangements of S100A11 contribute to signaling fidelity in Akt-dependent pathways.

Collectively, these results indicate that low expression of PCK1 mainly reduces H3K9me3 modification by restricting SAM generation, which enhances *S100A11* transcription and expression and aberrant activation of the PI3K/AKT pathway, subsequently promoting HCC malignant development and progression. These findings provide insights into the role and contribution of PCK1-mediated histone methylation in tumor suppression, implying that fluctuations in metabolism are intimately linked to dysregulation of epigenetic modifications. Targeting these pathways for SAM availability and suppression of oncogene S100A11 therefore represent potential applications in the clinical treatment of HCC.

## Methods

### Animal models.

*AlbCre*^+/–^ and *Pck1*^fl/fl^ (LKO) mice were generated from crosses between *AlbCre^+/–^* mice (Model Animal Research Center of Nanjing University, Nanjing, China) and *Pck1*^fl/fl^ mice on a C57BL/6 background (Mutant Mouse Resource & Research Centers; MMRRC:011950-UNC), and *AlbCre*^–/–^ and *Pck1*^fl/fl^ (WT) mice were used as controls. To induce HCC, 2-week-old mice were injected intraperitoneally with 50 mg/kg DEN, and, from 4 weeks of age once per week, with CCl_4_ (2 mL/kg) diluted in sterile olive oil for an additional 16 weeks. All mice were fed a diet containing 0.05% phenobarbital (PB). The *Pck1*-LKO + SAM group was administered SAM (100 mg/kg/d 4 days per week for 8 weeks) 32 weeks after DEN/CCl_4_/PB treatment, and *Pck1*-LKO + pSECC-sg*S100a11* mice were injected with pSECC-based *S100a11*-KO lentiviruses into the lateral tail vein at 8 weeks old.

### Cell lines and cell culture.

PLC/PRF/5, SK-Hep1, MHCC-97H, HEK-293, HEK-293T, and Hep1-6 cells were grown in DMEM (HyClone) with 10% FBS (Gibco) and 1% penicillin/ streptomycin (10,000 U/mL, HyClone) at 37°C in 5% CO_2_. SNU449 cells were grown in RPMI 1640 medium with 10% FBS and 1% penicillin/streptomycin (10,000 U/mL, HyClone).

### Plasmid construction.

PCR-amplified *S100A11* was cloned into pSEB-3Flag. PCR-amplified *AKT1*, ΔN truncated *AKT1* 108–480 amino acids, ΔC truncated *AKT1* 1–151 amino acids were separately cloned into pBu-3HA. PCR-amplified *PCK1* and *G309R* were separately cloned into pBu-3HA. All plasmids were constructed by standard molecular biology techniques and verified by DNA sequencing.

### Gene inactivation by the CRISPR/Cas9 system.

sgRNAs were used to KO *PCK1*, *PHGDH*, *SUV39H1*, or *S100A11* by the CRISPR/Cas9 system. KO cell clones were screened by Western blotting to verify the loss of PCK1, PHGDH, SUV39H1, or S100A11 expression. The oligo sequence information for sgRNAs used for KO cell generation is listed in the Supplemental Table 1.

### Adenovirus production.

The human cDNA fragment *PCK1* (NM_002591) or *G309R* (PCK1 mutation 925G>A) was cloned into the pAdTrack-TO4 vector (from Tong-Chuan He, University of Chicago, Chicago, Illinois, USA). Recombinant adenoviral AdPCK1, and AdG309R were constructed by the AdEasy system as described previously (19). The adenoviral AdGFP was used as a control.

### Generation of the pSECC lentiviruses.

*S100a11* sgRNA was cloned into the pSECC vector (a lentiviral vector encoding Cre and CRISPR components, Addgene plasmid 60820). HEK293T cells were cotransfected with 2.5 μg of pSECC-sgRNA plasmid and 5 μg of psPAX2 packaging plasmid and 7.5 μg of pMD2.G packaging plasmid (m/m/m = 1/2/3). Ten hours after transfection, the supernatant was replaced with a fresh culture medium. Seventy-two hours after transfection, the supernatant viruses were collected by centrifugation for 20 minutes (4,000*g*, 4°C). The precipitate was centrifuged for 12 hours (120,000*g*, 4°C) followed by dilution in PBS. Finally, RT-PCR was used for quantification.

### Western blot.

Cells and tissues were lysed with cold lysis buffer (Beyotime Biotechnology) containing 1 mM phenylmethanesulfonyl fluoride (Beyotime Biotechnology). Cell mixture was incubated on ice for 20 minutes with vortex, followed by sonication. Then, the mixture was centrifuged for 20 minutes (14,000*g*, 4°C). Supernatant protein was quantified using BCA Protein Assay Kit (Beyotime Biotechnology). Protein extracts were separated by 10% or 12% SDS/PAGE and transferred to PVDF membranes (Millipore). Membranes were blocked in 5% nonfat milk in TBST for 1.5 hours and then incubated with the indicated antibodies (Supplemental Table 2) overnight at 4°C.

### Metabolite extraction and analysis.

The cells were washed twice with ice-cold PBS and then quickly frozen in liquid nitrogen. Metabolites were extracted with 200 μL cold methanol/acetonitrile/water (2:2:1, v/v/v) and incubated on ice for 20 minutes. The mixture was sonicated and vortexed and then centrifuged for 20 minutes (14,000*g*, 4°C). Finally, the supernatant was dried using a vacuum centrifuge. For LC-MS analysis, the samples were redissolved in 60 μL methanol.

To extract metabolites from serum samples, 120 μL cold extraction solvent (1:1, methanol/acetonitrile, v/v) was added to 30 μL of the serum sample. After vortexing, the samples were incubated on ice for 20 minutes, centrifuged for 20 minutes (14,000*g*, 4°C), and 100 μL supernatant was subjected to LC/MS analysis.

To extract metabolites from tissue samples, 100 μL cold methanol/acetonitrile/water (2:2:1, v/v/v) was added to 60 mg powderized tissues, followed by vortexing. The samples were incubated on ice for 20 minutes and then centrifuged for 20 minutes (14,000*g*, 4°C). Finally, 100 μL supernatant was subjected to LC-MS analysis.

### Untargeted metabolomics.

For untargeted metabolomics of PCK1-overexpressing (AdGFP as control) SK-Hep1 cells, extracts were analyzed using an UHPLC (1290 Infinity LC, Agilent Technologies) coupled to quadrupole time-of-flight (AB Sciex TripleTOF 6600) at Applied Protein Technology.

### RNA m6A dot blotting.

mRNA was isolated from total RNA using the mRNA Purification Kit (TIANGEN) following the manufacturer’s instructions. mRNA was heated at 65°C for 15 minutes and cooled immediately on ice for 5 minutes. mRNA samples (500, 200, and 100 ng) were spotted on the Hybond-N+ membrane (GE Healthcare). After UV cross-linking, the membrane was washed with 1× TBST buffer and blocked with 5% skim milk. Then, the blot was incubated with the anti-m6A antibody (1:1,000, Abcam) overnight at 4°C. The membrane was washed, incubated with an anti-mouse antibody, and washed again. Finally, the membrane was exposed to Hyperfilm ECL, and images were acquired. Methylene blue interacted with mRNA and was used as the loading control.

### Proliferation assays.

Cells were plated at a concentration of 800 cells per well in triplicate into 96-well plates for 6 days. For cell growth assays with chemical treatment, fresh chemicals were added to the media every 2 days. Cell growth rates were analyzed daily by cell counting with IncuCyte ZOOM software (ESCO).

### Colony formation.

Cells were seeded into 6-well plates at a density of 1,000 cells/well. The cells were cultured for 7–12 days, fixed with 4% paraformaldehyde for 15 minutes, and stained with 0.1% crystal violet. The colonies were photographed and counted by Image-Pro Plus software (version 6.0, Media Cybernetics Inc.).

### Transwell migration assay.

Cell invasion was evaluated in 24-well Transwell chambers (Corning). In the Transwell chambers, the upper and lower culture compartments of each well are separated by a polycarbonate membrane (pore size, 8 μm). Total of 2 × 10^4^ cells that had been treated as indicated were placed into the upper chambers with 200 μL serum-free medium, and 800 μL medium supplemented with 10% FBS was added to the lower chambers. The invasive cells were stained with crystal violet and quantified (3 random 200 × fields per well) under Axio Imager A2 (ZEISS). The invasive potential of cells was normalized with the cell proliferation rate. The invasive potential of cells was calculated as follows: numbers of invading cells = numbers of cells in Transwell chambers × (1 – ((cell numbers after 24 hours of proliferation – cell numbers after 0 hours of proliferation)/cell numbers after 0 hours of proliferation)). Fold change was calculated by setting the control group value to 1.

### Wound-healing assay.

Cells were cultured in 96-well plates, and wounds were created using WoundMaker (ESSEN) on the cell surface. The wound areas were recorded by the IncuCyte ZOOM software (ESCO).

### H3K9 methyltransferase activity tests.

Nuclear proteins were extracted using a Nuclear and Cytoplasmic Protein Extraction Kit (P0027, Beyotime) according to the manufacturer’s instructions. The enzymatic activity of HMT for H3K9 was detected using the EpiQuik Histone Methyltransferase Activity Assay Kit (P-3003, EpiQuik) according to the manufacturer’s instructions.

### ^13^C-labeled glutamine tracing.

For ^13^C-glutamine labeling, SK-Hep1 cells were infected with AdPCK1 or AdGFP, followed by culturing in DMEM (no glucose and no glutamine, Gibco, A1443001) supplemented with 4.5 mg/mL unlabeled glucose, 10% dialyzed FBS, and 0.3 mg/mL U-[^13^C]-glutamine (Cambridge Isotope Laboratories Inc., catalog CLM-1822-H-0.1) for 24 hours. For ^13^C-pyruvate labeling, cells were cultured in DMEM (no sodium pyruvate, Gibco, 11965) supplemented with 1 mM U-[^13^C]-pyruvate (Cambridge Isotope Laboratories Inc., catalog CLM-2440-0.1) for 24 hours. For ^13^C-methionine labeling, cells were cultured in DMEM (no methionine, Gibco, 21013024) supplemented with 0.2 mM U-[^13^C]-methionine (Cambridge Isotope Laboratories Inc., catalog CLM-893-H-0.05) for 6 hours. The cells were then washed with cold PBS, and the metabolites were extracted using 80% cold methanol. LC-MS was performed by LipidALL Technologies, and metabolite abundance was expressed relative to the internal standard, according to standard protocols.

### RNA-Seq and ChIP-Seq.

Parental or PCK1-KO PLC/PRF/5 cells (1 × 10^7^) were harvested, and total RNA was isolated using TRIzol reagent (Invitrogen) following the manufacturer’s protocol. High-quality (Agilent Bioanalyzer, RIN > 7.0) total RNA was used for the preparation of sequencing libraries using the Illumina TruSeq Stranded Total RNA/Ribo-Zero Sample Prep Kit. Paired-end sequencing was carried out on an Illumina Novaseq 6000 at LC-BIO Bio-tech Ltd., following the manufacturer’s recommended protocol. Differentially expressed mRNAs and genes were selected using the R package with log_2_ (fold change) values of ≥1 or log_2_ (fold change) values of ≤–1 and with statistical significance of *P* < 0.05.

ChIP-Seq was performed on 1 × 10^7^ parental or PCK1-KO PLC/PRF/5 cells. A cross-linked cell pellet was prepared, followed by ChIP-Seq using an antibody anti-H3K9me3 (ab8898; Abcam). The sequence reads generated by Illumina sequencing were mapped to the genome using the Bowtie2 algorithm with the default settings. Peak calling was performed using MACS2 (macs2 callpeak -g hs -q 0.05 --broad --broad -cutoff 0.05; other settings were set to default parameters). BigWig files were imported into the Integrative Genomics Viewer (IGV, v2.7.0) for the visualization of specific loci.

### Quantitative RT-PCR.

Total RNA was extracted with TRIzol reagent (Invitrogen). RT-PCR reactions were performed with the PrimeScript RT Reagent Kit (RR047A, TaKaRa). Real-time PCR was performed using SYBR Green qPCR Master Mix (Bio-Rad) and the CFX Connect Real-time PCR Detection System (Bio-Rad). β-Actin expression was used for normalization and comparative2^–ΔΔCt^ methods were used for quantification of gene expression. The sequence information for q-PCR primer pairs used for gene expression analysis is listed in the Supplemental Table 1.

### ChIP assay.

6 × 10^6^ cells were harvested and cross-linked with 1% formaldehyde in DMEM medium at 37°C for 10 minutes. Cell lysate was sonicated and subjected to immunoprecipitation using anti-H3K9me3 (ab8898, Abcam) antibody or control IgG overnight at 4°C. After extensive wash, immunoprecipitated DNA was amplified by real-time PCR. The data analysis was expressed as percentages of the input DNA. Anti-rabbit IgG was used as a negative control. Primers used to amplify the promoter regions of *S100A11* are shown in Supplemental Table 1.

### Immunofluorescent cytochemistry.

Cells cultured on coverslip were fixed in 4% paraformaldehyde for 15 minutes. After washing in PBS 3 times, fixed cells were permeabilized with 0.5% Triton X-100 for 10 minutes, washed in PBS, and then blocked with 10% goat serum. For 5mc and 5hmc staining, cells on coverslips were fixed with ice-cold 70% ethanol for 8 minutes at room temperature and then washed in PBS. To denature the DNA, coverslips were incubated with 2 M HCl for 30 minutes at room temperature. Then, specimens were permeabilized with Triton X-100 for 10 minutes and blocked with 10% goat serum for 40 minutes at room temperature. Cells were incubated with indicated primary antibody at 4°C overnight. After washing 3 times with PBS, cells were incubated with the secondary antibody (goat anti-rabbit IgG/TRITC, goat anti-mouse IgG/TRITC, or goat anti-mouse IgG/FITC) for 45 minutes at 37°C. After washing 3 times with PBS, nuclei were counterstained with DAPI (10236276001, Roche Diagnostics GmbH). The coverslips were mounted onto glass slides with an antifade solution and visualized under a laser-scanning confocal microscope (Leica TCS SP8).

### Immunoprecipitation.

Cells were lysed in lysis buffer (150 mM NaCl, 50 mM Tris, pH 7.5, 0.5% NP-40) containing 1 mM PMSF, 1 mM phosphatase inhibitors (Beyotime Biotechnology), and protease inhibitors (Roche) and incubated on ice for 30 minutes. Cell lysates were centrifuged for 10 minutes (120,000*g*, 4°C). Equal amounts (50 μg) of proteins from each sample were boiled in loading buffer as input protein. The rest of lysates were incubated with the primary antibody for 6–8 hours at 4°C. Protein A/G magnetic bead was added to the incubation mixture for an additional 2–3 hours to pull down antibody-protein complexes. The immunoprecipitates were collected and washed 3 times with PBST buffer before being resolved by SDS-PAGE.

### Orthotopic HCC, subcutaneous HCC, and lung metastasis models.

Male 4- to 6-week-old BALB/c nude mice were used for the orthotopic HCC model: PLC/PRF/5 cells (1 × 10^6^, parental, *n* = 6; S100A11 KO, *n* = 6; PCK1 KO, *n* = 6; or PCK1/S100A11–double KO, *n* = 6) were mixed in 50% volume of 50 μL Matrigel (356234, BD Biosciences) and then implanted into the left liver lobe. Eight weeks after implantation, all mice were sacrificed.

For the subcutaneous HCC model, MHCC-97H cells (1 × 10^6^), which include AdGFP- (*n* = 6), AdPCK1- (*n* = 6), or AdPCK1+ S100A11-overexpression cells (*n* = 6), were resuspended in 100 μL PBS and injected into the right posterior flanks of 4- to 6-week-old BALB/c nude mice. All mice were euthanized 24 days after the subcutaneous injection. The tumors were excised, photographed, and weighed.

For the lung metastasis model, SNU449 cells (parental, *n* = 6; S100A11 KO, *n* = 6; PCK1 KO, *n* = 6; or PCK1/S100A11–double KO, *n* = 6) were suspended in PBS (1 × 10^6^/200 μL) and injected into the lateral tail veins of male BALB/c nude mice. The mice were sacrificed after 8 weeks.

### IHC.

Primary antibodies against PCK1 (1:600), H3K9me3 (1:600), S100A11 (1:600), and p-AKT (Ser473) (1:400) were used for immunohistochemical staining of formalin-fixed paraffin-embedded human or mouse liver tissues. Subsequently, the slides were incubated with secondary anti-rabbit IgG (ZSGB-BIO) and visualized using 3, 3′-diaminobenzidine (ZSGB-BIO). The stained slides were scanned with a Pannoramic Scan 250 Flash or MIDI system, and images were acquired using Pannoramic Viewer software (version 1.15.2, 3DHistech).

### TCGA database analysis.

Data sets for genes from TCGA data portal were accessed and downloaded from the cBioPortal for Cancer Genomics (https://www.cbioportal.org/). mRNA expression correlation was evaluated in the human liver cancer TCGA database. Kaplan-Meier survival curves were plotted by R package “survminer.”

### Statistics.

Graphical representations and statistical analyses included 2-tailed unpaired Student’s *t* test, 2-tailed paired Student’s *t* test, 1-way ANOVA, 2-way ANOVA, Pearson’s correlation coefficient, and log-rank test, calculated using GraphPad Prism 6 or Microsoft Excel 2019. *P* values of less than 0.05 were determined to be significant. For experiments involving quantitative analysis, we used independent biological replicates with similar results, as indicated in the figure legends. For tumor analysis, at least 6 independent tumors from each group were used for the analysis. The experiments were not randomized, with the exception that the mice were randomly grouped before treatments. Samples were allocated to their experimental groups according to their predetermined type, and investigators were not masked to allocation during the experiments and outcome assessment.

### Study approval.

Primary HCC tissue samples and paired adjacent normal tissue samples were obtained from the Second Affiliated Hospital of Chongqing Medical University between 2015 and 2018, with approval from the Institutional Review Board of Chongqing Medical University. All the tissue samples were well preserved at –180°C until they were used in further experiments. Written informed consent was obtained from all patients in accordance with a protocol approved by the Second Affiliated Hospital of Chongqing Medical University.

Mice were housed in a specific pathogen–free facility under standard conditions. All animal experiments were performed under the guidelines of the Institutional Animal Care and Use Committee at Chongqing Medical University. All animal procedures were also approved by the Research Ethics Committee of Chongqing Medical University.

### Data availability.

RNA-Seq and ChIP-Seq data sets were deposited in BioProject (accession PRJNA818729). Bisulfite sequencing (RRBS) has been deposited to the Gene Expression Omnibus database (GEO, accession GSE221725). See complete unedited blots in the supplemental material. Values for all data points in graphs are reported in the supporting data values file.

## Author contributions

NT, KW, and AH conceived the study and designed the experiments. DG, RL, XS, and HD conducted most experiments and data analysis. CC provided assistance with experiments. JX and YL performed animal experiments. QG and ZL constructed plasmids and generated cell lines. DG, NT, and KW prepared the manuscript, with feedback from all authors.

## Supplementary Material

Supplemental data

Supporting data values

## Version 1 05/11/2023

In-Press Preview

## Version 2 07/03/2023

Electronic publication
