# Supplementary material for: Gluconeogenic enzyme PCK1 supports S-adenosylmethionine biosynthesis and promotes H3K9me3 modification to suppress hepatocellular carcinoma progression
Source: J Clin Invest. 2023 Jul 3;133(13):e161713. doi: 10.1172/JCI161713 (PMC10313362; doi:10.1172/JCI161713)
Supplement: Supplemental data [file jci-133-161713-s025.pdf]

Supplemental data

Gluconeogenic enzyme PCK1 supports S-adenosylmethionine biosynthesis and promotes H3K9me3 modification to suppress hepatocellular carcinoma progression

Dongmei Gou[1, #], Rui Liu[1, #], Xiaoqun Shan[1, #], Haijun Deng[1, #], Chang Chen[2, #], Jin Xiang[1], Yi Liu[1], Qingzhu Gao[1], Zhi Li[3], Ailong Huang[1,*], Kai Wang[1,*], and Ni Tang[1,*]

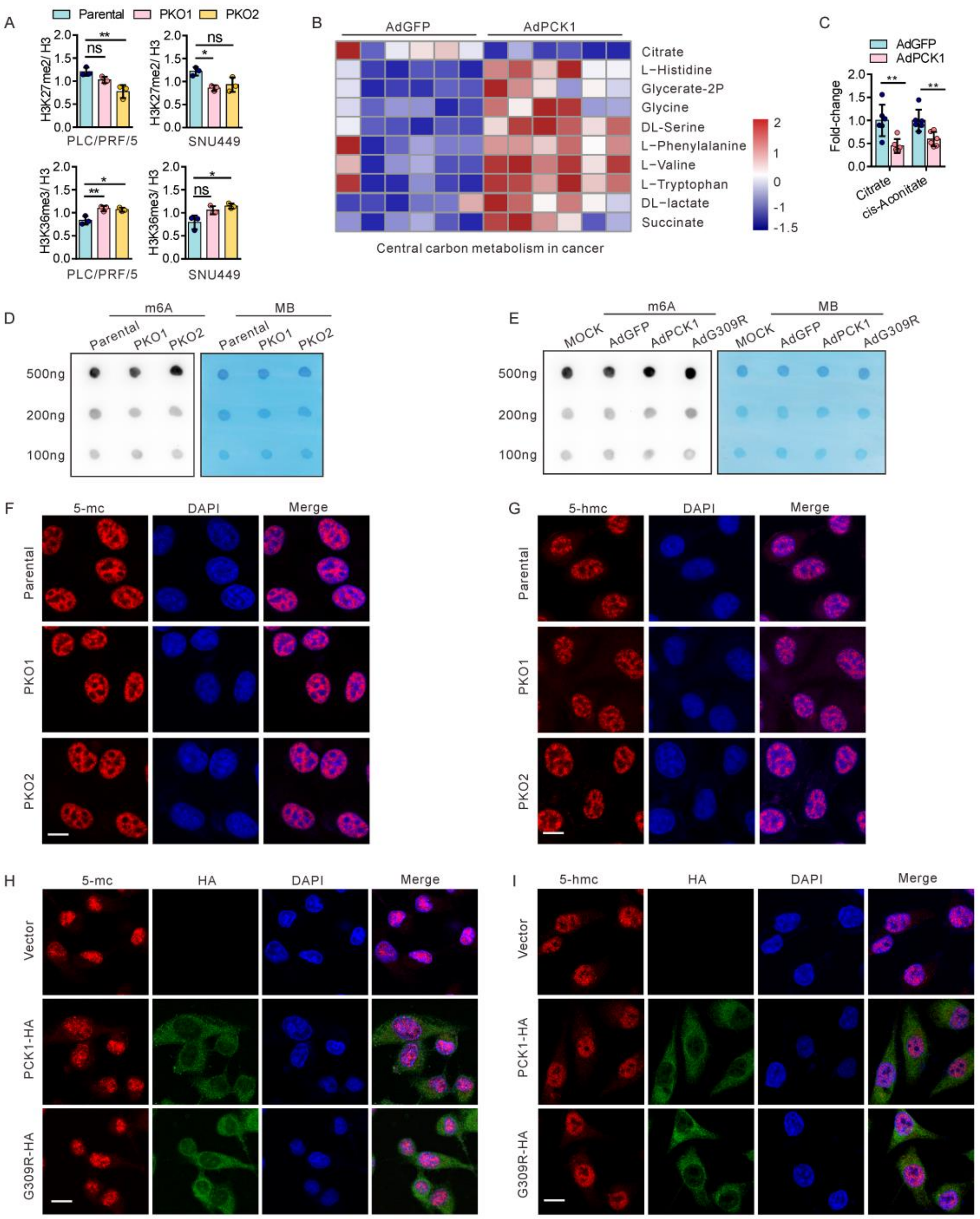

Supplemental Figure 1. PCK1 upregulates H3K9me3 and provides methyl donors by enhancing SSP flux. (**A**) Densitometric analysis of H3K27me2 (representative blots in Figure 1A) and H3K36me3 (representative blots in Figure 1A) in PCK1-knockout PLC/PRF/5 cells (PKO cells) and PCK1-knockout SNU449 cells (PKO cells); histone H3 was used as loading control (n=3). (**B**, **C**) Heatmap showing central carbon metabolites in cancer (B) and fold-changes in intermediate metabolites of the TCA cycle (C) in SK-Hep1 cells overexpressing WT PCK1 (n = 6 biologically independent samples). (**D**,

E) Dot blot analyses with an anti-m^{6}A antibody, and MB (methylene blue) staining served as the loading control. (**F**, **G**) Immunofluorescent images of 5-mc (F) and 5-hmc (G) in PCK1-knockout PLC/PRF/5 cells (PKO cells). (**H**, **I**) Immunofluorescence images for 5-mc (H) and 5-hmc (I) in SK-Hep1 cells transfected with PCK1-overexpressing plasmid. Scale bars: 15 μm (F-I). Data are shown as the mean ± SEM. Statistical analysis was performed using 2-tailed unpaired Student's t test (C) or 1-way ANOVA with Tukey's test (A); *$P < 0.05$, **$P < 0.01$.

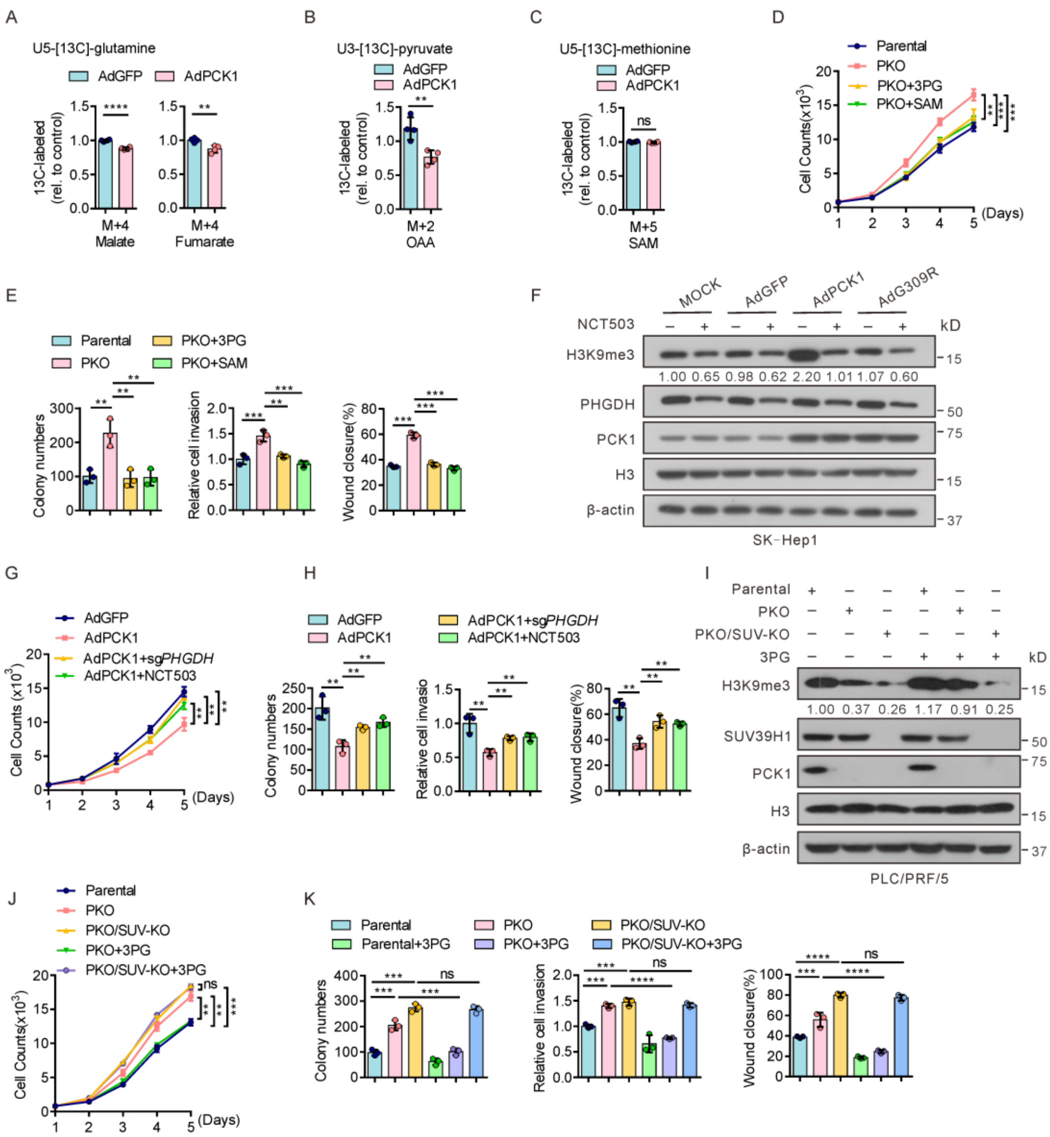

Supplemental Figure 2. PCK1 enhances H3K9me3 modification by SAM via SSP and SUV39H1. (**A**) LC-MS profiles of m+4 Malate and m+4 Fumarate, respectively, after PCK1-OE cells were incubated with U-[^{13}C]-glutamine for 24 h (n = 4 biologically independent samples). (**B**) LC-MS profiles of m+2 OAA, after PCK1-OE cells were incubated with U-[^{13}C]-pyruvate for 24 h (n = 4 biologically independent samples). (**C**) LC-MS profiles M+5 SAM after PCK1-OE cells were incubated with U-[^{13}C]-methionine for 6 h (n = 4 biologically independent samples). (**D**, **E**) PKO cells were treated with

or without 3PG (0.75 mM) and SAM (50 μM) for 24 h; cell growth curves (D) (n = 3 technical replicates), colony formation assays, transwell assays, and wound scratch assays (E) (n = 3 biologically independent samples) shown as indicated. (**F**) Western blot detection of H3K9me3 modification in SK-Hep1 cells treated with the inhibitor NCT503 (30 μM) for 24 h, after which cells were infected with WT PCK1 for 24 h. (**G, H**) SK-Hep1 cells were knocked down of PHGDH or treated with the inhibitor NCT503 (30 μM) for 24 h, after which cells were infected with WT PCK1 for 24 h; cell growth curves (G) (n = 3 technical replicates), colony formation assays, transwell assays, and wound scratch assays (H) (n = 3 biologically independent samples) shown as indicated. (I-K) PKO or PKO/SUV-KO cells were supplemented with or without 3PG (0.75 mM) for 24 h; western blot detection of indicated protein (I), cell growth curves (J) (n = 3 technical replicates), colony formation assays, transwell assays, and wound scratch assays (K) (n = 3 biologically independent samples) shown as indicated. Data are shown as the mean ± SEM. Statistical analysis was performed using 2-tailed unpaired Student's t test (A-C), 1-way ANOVA with Tukey's test (E, H, K) or 2-way ANOVA with Bonferroni's test (D, G, J); ***P* < 0.01, ****P* < 0.001, *****P* < 0.0001.

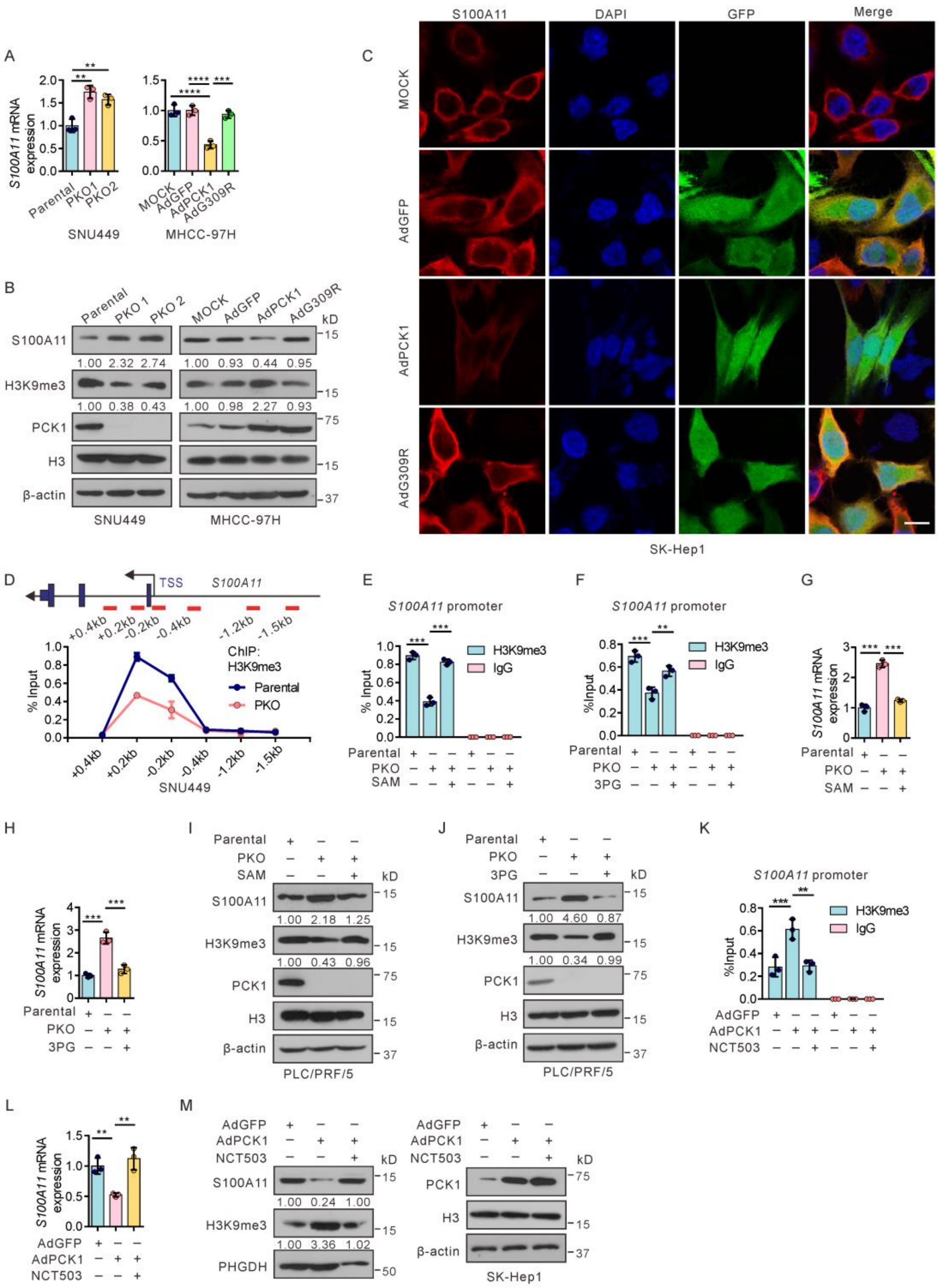
A
S100A11 mRNA expression
Parental
PKO1
PKO2
SNU449
MOCK
AdGFP
AdPCK1
AdG309R
MHCC-97H
B
S100A11
H3K9me3
PCK1
H3
β-actin
kD
1.00 2.32 2.74
1.00 0.93 0.44 0.95
1.00 0.38 0.43
1.00 0.98 2.27 0.93
SNU449
MHCC-97H
C
S100A11
DAPI
GFP
Merge
MOCK
AdGFP
AdPCK1
AdG309R
SK-Hep1
D
TSS
S100A11
+0.4kb
+0.2kb
-0.2kb
-0.4kb
-1.2kb
-1.5kb
% Input
ChIP: H3K9me3
Parental
PKO
SNU449
E
S100A11 promoter
% Input
H3K9me3
IgG
Parental
PKO
SAM
F
S100A11 promoter
%Input
H3K9me3
IgG
Parental
PKO
3PG
G
S100A11 mRNA expression
Parental
PKO
SAM
H
S100A11 mRNA expression
Parental
PKO
3PG
I
Parental
PKO
SAM
kD
S100A11
H3K9me3
PCK1
H3
β-actin
1.00 2.18 1.25
1.00 0.43 0.96
PLC/PRF/5
J
Parental
PKO
3PG
kD
S100A11
H3K9me3
PCK1
H3
β-actin
1.00 4.60 0.87
1.00 0.34 0.99
PLC/PRF/5
K
S100A11 promoter
%Input
H3K9me3
IgG
AdGFP
AdPCK1
NCT503
L
S100A11 mRNA expression
AdGFP
AdPCK1
NCT503
M
AdGFP
AdPCK1
NCT503
kD
S100A11
H3K9me3
PHGDH
1.00 0.24 1.00
1.00 3.36 1.02
PCK1
H3
β-actin
SK-Hep1

Supplemental Figure 3. PCK1 suppresses S100A11 by increasing SAM-dependent H3K9me3 occupancy. (**A**) qPCR analysis of *S100A11* expression in SNU449 cells (PKO) (left) and PCK1-OE MHCC97H cells (right) (n = 3 technical replicates). (**B**) western blot detection of S100A11 expression in SNU449 cells (PKO) (left) and PCK1-OE MHCC97H cells (right). (**C**) Immunofluorescence images of S100A11 in PCK1-OE cells. (**D**) ChIP-qPCR was performed to evaluate the enrichment of H3K9me3 in different promoter regions of *S100A11* in SNU449 cells (PKO cells) (n = 3 technical replicates). (**E**-**J**) PLC/PRF/5 cells (PKO cells) were supplemented with SAM (50 μM) or 3PG (0.75 mM) respectively for 24 h, followed by ChIP-qPCR analysis of H3K9me3 enrichment at the *S100A11* promoter (E, F) (n = 3 technical replicates), *S100A11* mRNA by qPCR (G, H) (n = 3 technical replicates), and western blot detection of S100A11 expression (I, J). (**K**-**M**) SK-Hep1 cells were infected with WT PCK1 for 24 h, after which cells in the presence or absence of the inhibitor NCT503 (30 μM) for 24 h, followed by ChIP-qPCR analysis of H3K9me3 enrichment at the *S100A11* promoter (K) (n = 3 technical replicates), detection of *S100A11* mRNA by qPCR (L) (n = 3 technical replicates), and western blot detection of S100A11 protein expression (M). Data are shown as the mean ± SEM. Statistical analysis was performed using 1-way ANOVA with Tukey's test (A, E-H, K, L); ***P* < 0.01, ****P* < 0.001, *****P* < 0.0001.

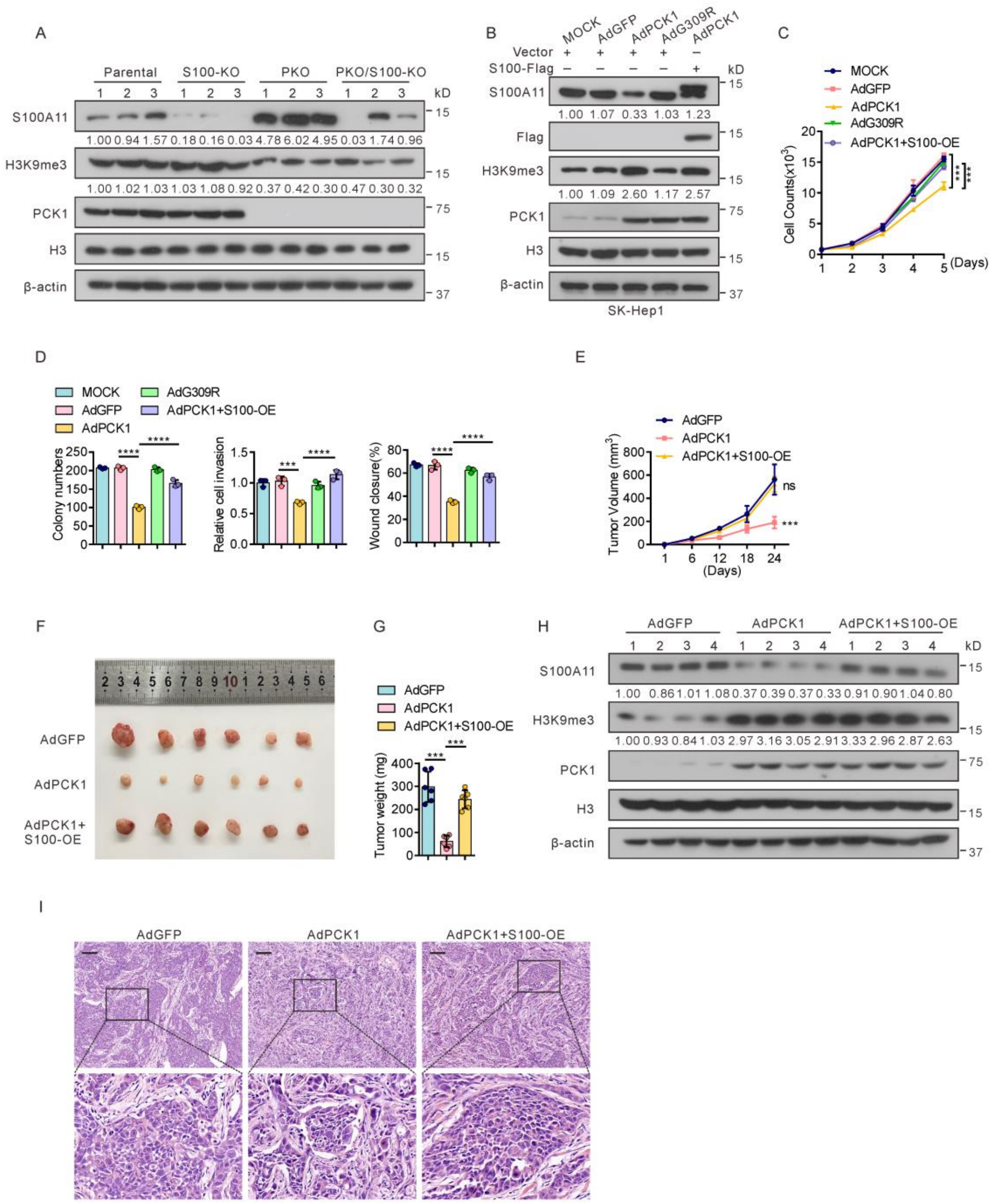

Supplemental Figure 4. PCK1 deficiency induces HCC cell proliferation, migration, and tumorigenesis via S100A11. (**A**) Western blot of the indicated proteins or modifications in liver tumors. (**B**-**D**) PCK1-OE cells transfected with S100A11-overexpressing plasmid for 36 h, followed by western blotting of the indicated proteins (B), cell proliferation assays (C) (n = 3 technical replicates), colony formation assays, transwell assays, and wound-healing assays (D) (n = 3 biologically independent samples) were performed as indicated. (**E**-**I**) Tumor volume (E) (n = 6 mice per group), gross

image (F), tumor weight (G) (n = 6 mice per group), expression of the indicated proteins (H), and H&E staining (I) in subcutaneous xenograft models. Scale bar: 100 μm (I). Data are shown as the mean ± SEM. Statistical analysis was performed using 1-way ANOVA with Tukey's test (D, G) or 2-way ANOVA with Bonferroni's test (C, E); ***$P < 0.001$, ****$P < 0.0001$.

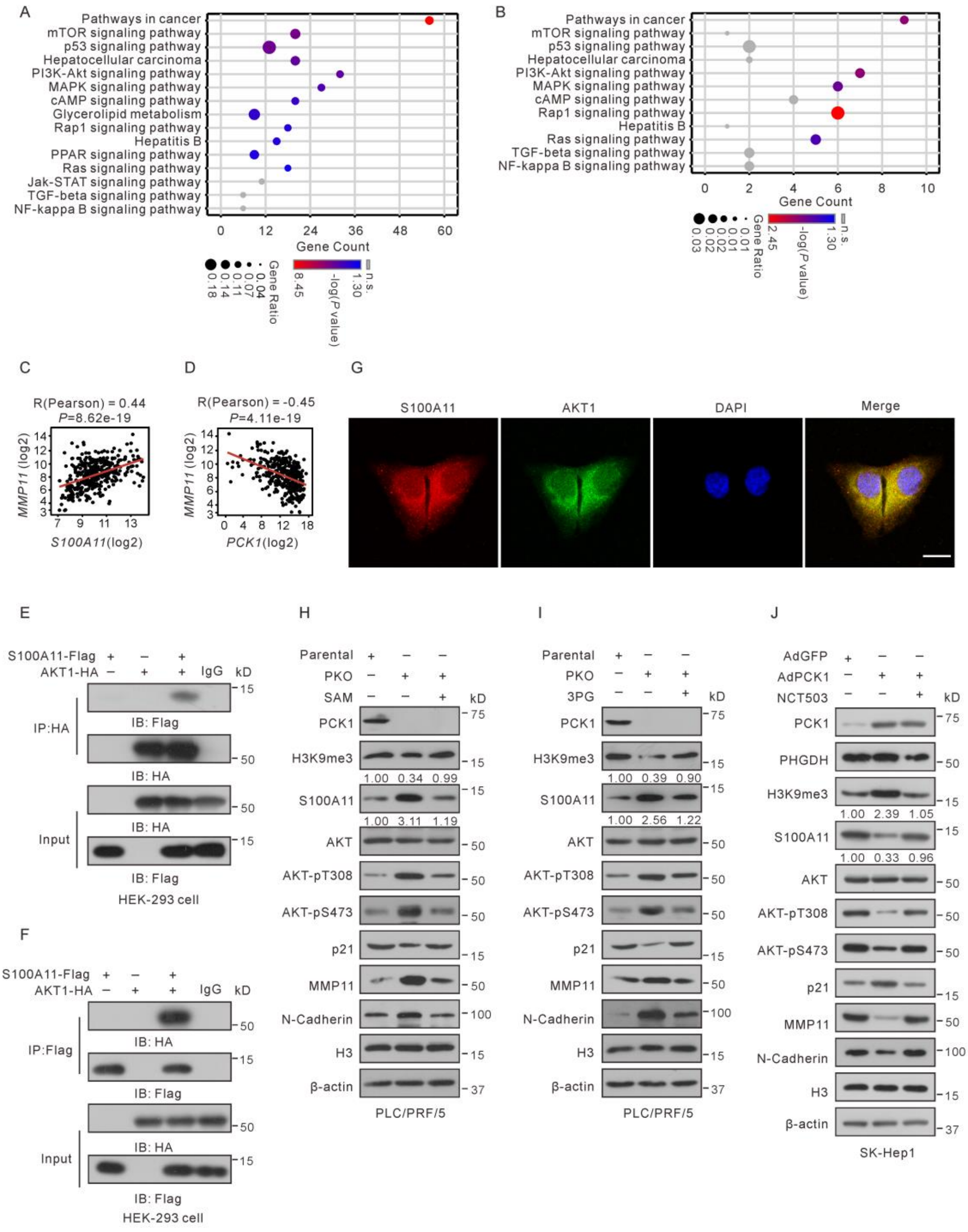

Supplemental Figure 5. Loss of PCK1 activates PI3K/AKT signaling through S100A11. (**A**, **B**) Enriched pathways of differentially expressed genes between PCK1-KO and parental PLC/PRF/5 cells revealed by RNA-seq analysis (A) (n = 4 biologically independent samples) and ChIP-seq (B). (**C**, **D**) Correlations of *PCK1* and *MMP11* (C), *S100A11* and *MMP11* (D) mRNA expression in HCC patient samples from TCGA database. (**E**, **F**) Co-IP of AKT1-HA and S100A11-Flag was examined using an anti-HA antibody (E) or an anti-Flag antibody (F) in 293 cells. (**G**) Subcellular co-localization of

AKT1 and S100A11 in PKO cells was determined by immunofluorescence staining. (**H**, **I**) PKO cells were treated with or without SAM (50 μM) (H) or 3PG (0.75 mM) (I) for 24 h, and then the indicated proteins or modifications were detected by western blot. (**J**) SK-Hep1 cells were infected with AdPCK1 for 24 h in the presence or absence of inhibitor NCT503 (30 μM), followed by western blotting of the indicated proteins. Scale bars: 15 μm (G). Data are shown as the mean ± SEM. Statistical analysis was performed using log-rank test (A, B).

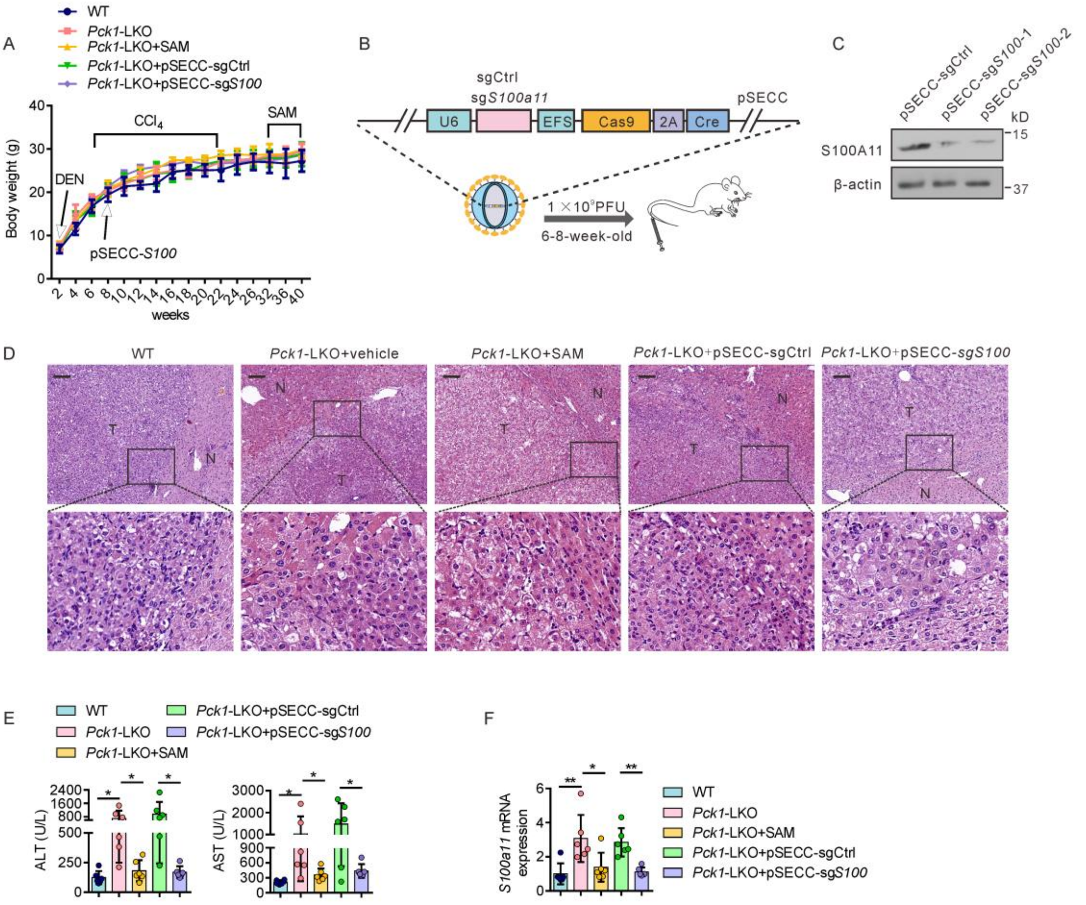

Supplemental Figure 6. Reductive H3K9me3 modification at *S100a11* promotes DEN/CCl_4/PB-induced hepatocellular carcinogenesis in *Pck1*-knockout mice. (**A**) Line chart of the body weights in the WT, *Pck1*-LKO+vehicle, *Pck1*-LKO +SAM, *Pck1*-LKO +pSECC-sgCtrl, and *Pck1*-LKO +pSECC-sg*S100a11* groups (n = 6 mice per group). (**B**) Schematic diagram of pSECC-sg*S100a11* lentiviruses packaged and injected into the tail vein of *Pck1*-LKO. (**C**) Western blot showing that S100A11 was silenced after infection with pSECC-sg*S100a11* supernatant viruses for 48 h in Hep1-6 cells.

(**D**) H&E staining of the mouse liver tissue. (**E**) ALT (left) and AST (right) levels in mouse serum samples (n = 6 mice per group). (**F**) qPCR analysis of *S100a11* (n = 6 mice per group). Scale bars: 100 μm (D). Data are shown as the mean ± SEM. Statistical analysis was performed using 1-way ANOVA with Tukey's test (E, F) or 2-way ANOVA with Bonferroni's test (A); *$P < 0.05$, **$P < 0.01$.

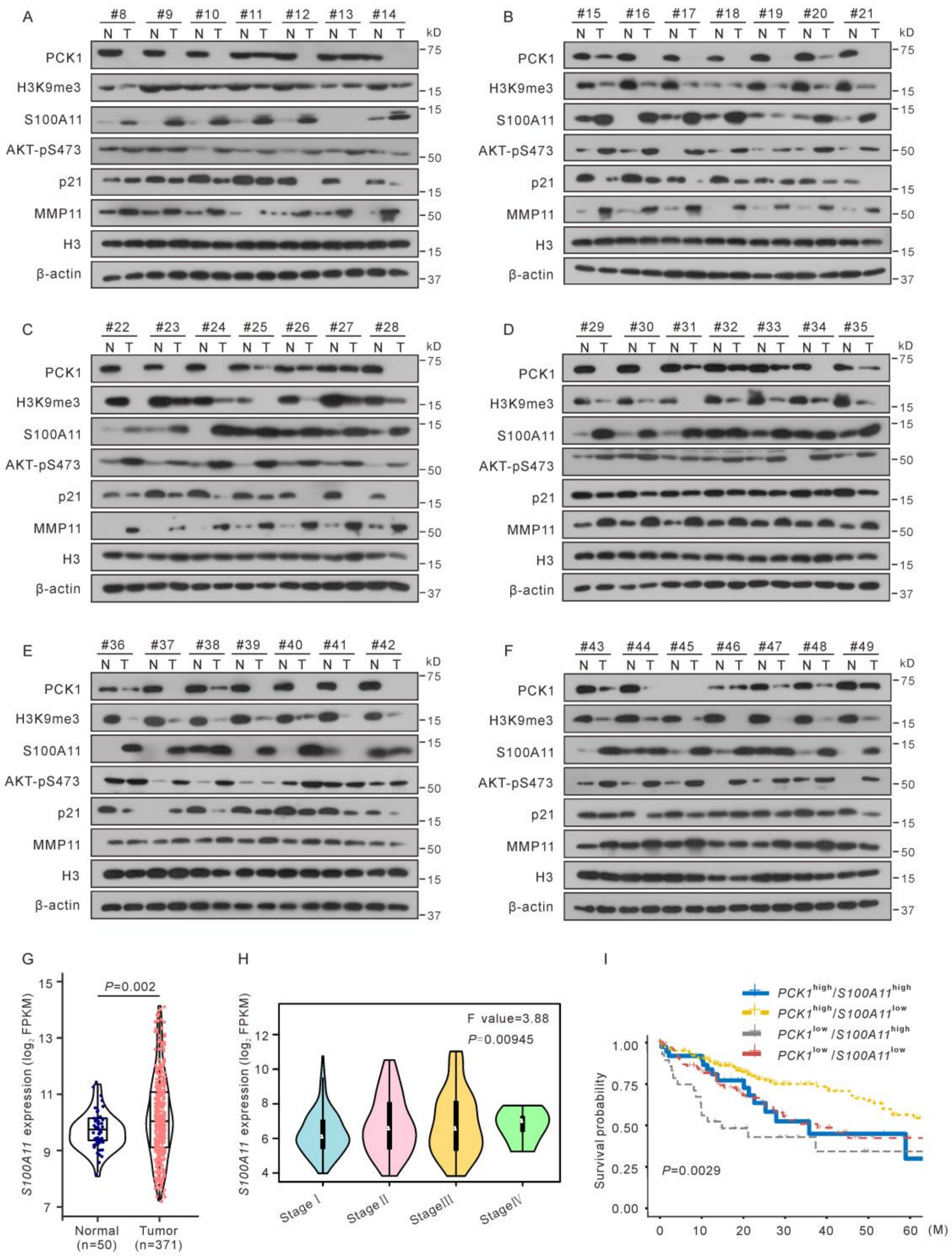

Supplemental Figure 7. Correlation between PCK1, H3K9me3, and S100A11 expression in HCC specimens. (**A**-**F**) Protein or modification levels in representative human HCC specimens and the surrounding non-tumorous tissues were measured by western blot analysis. (**G**, **H**) Dot plot showing the relative fold change of *S100A11* mRNA expression between HCC (n=371) and non-tumoural tissues (n=50) (TCGA database) (G). Correlation between *S100A11* mRNA levels and tumor stage in HCC patients (GEPIA database) (H). (**I**) Kaplan-Meier plots showed high expression of *PCK1* and

low expression of *S100A11* significantly predicted better survival. Data are shown as the mean ± SEM. Statistical analysis was performed using 2-tailed unpaired Student's t test (G), 1-way ANOVA with Tukey's test (H), or Gehan–Breslow–Wilcoxon test (I).

Supplemental Table S1. Primer sequences used in this study

RT-qPCR primers			
Species	Gene	Forward (5’-3’)	Reverse (5’-3’)
human	*S100A11*	TGACCGCATGATGAAGAAACT	GACAGAAAGGCTGGAAGGAAA
mouse	*S100A11*	CTCCTGTCCCAGCCACCG	GGAGAGTTGAGTGTTGTTTCCATC
human	*PIK3R5*	AGGAAGAGGAGGAGGAGGAGGT	TAGCCGCTGTCCATGCCATCA
human	*AKT1*	GGCAAGGGCACTTTCGG	AGAGGCGGTCGTGGGTC
human	*AKT2*	AGTTTAGCTTTTGTGGGTTTGC	GGTTGGGCTGGCGTGA
human	*AKT3*	TTGTCGAGAGAGCGGGTGTTCT	ATGGTGGCTGCATCTGTGATCC
human	*p21*	GACACCACTGGAGGGTGACT	CAGGTCCACATGGTCTTCCT
human	*MMP11*	AGGCAGGGACTACTGGCGTTT	AGCCTTCCAGAGCCTTCACCTT
ChIP-qPCR Primers			
Species	Gene	Forward (5’-3’)	Reverse (5’-3’)
human	*S100A11-ChIP-a*	TCTTTCATTTGCGTCTACTCTGC	GCCTTTCCTGTTTCATAAGATTG
human	*S100A11-ChIP-b*	TTTTCGGATTTTGTCTTTGTGG	TGGGGATTATTGTCAAGGGAC
human	*S100A11-ChIP-c*	TTTGCCTATCTAACCTAAATGCC	TTATAAGTTTTGCACAGTAGTCTTCG
human	*S100A11-ChIP-d*	AGACTGCTTAAATCGCTGGAGA	TCAACCCACGCCCTTCC
human	*S100A11-ChIP-e*	GCCGCTCCCAGCCACA	CCATCCCTTCTTCCCCAGTT
human	*S100A11-ChIP-f*	TAGGGTAGCAGCGGAGGTT	AGGGGCTGGAGGCAGTG
CRISPR/Cas9 KO Target Primers			
Species	Gene	Forward (5’-3’)	Reverse (5’-3’)
human	lentiCRISPR-v2	GGTTTATTACAGGGACAGCAG	ACACGACATCACTTTCCCAG
human	*PCK1(sgRNA-1)*	CACCGGCTGAAGAAGTATGACAAC	AAACGTTGTCATACTTCTTCAGCC
human	*PCK1(sgRNA-2)*	CACCGTAGGCTGTCCAGGCTTCCC	AAACGGGAAGCCTGGACAGCCTAC
human	*SUV39H1(sgRNA-1)*	CACCGCGTGTGTTGCAAGTCTTCT	AAACAGAAGACTTGCAACACACGC
human	*SUV39H1(sgRNA-2)*	CACCGAGACTGACTTGACCAATGG	AAACCCATTGGTCAAGTCAGTCTC
human	*SUV39H1(sgRNA-3)*	CACCGTCTTCTTGGAATCAGCTGC	AAACGCAGCTGATTCCAAGAAGAC

human	*S100A11(sgRNA-1)*	CACCGAGAACTAGCTGCCTTCACAA	AAACTTGTGAAGGCAGCTAGTTCTC
human	*S100A11(sgRNA-2)*	CACCGCTGTCTTCCAGAAGTATGC	AAACGCATACTTCTGGAAGACAGC
human	*S100A11 (sgRNA-3)*	CACCGAAGCTTAGGAACTCTGTCT	AAACAGACAGAGTTCCTAAGCTTC
human	*PHGDH (sgRNA-1)*	CACCGGTTATGAAGTAAGTCATGG	AAACCCATGACTTACTTCATAACC
human	*PHGDH (sgRNA-2)*	CACCGACATCAGCGGTCACCTTGG	AAACCCAAGGTGACCGCTGATGTC
human	*PHGDH (sgRNA-3)*	CACCGCGACGGCTTCGATGAAGGA	AAACTCCTTCATCGAAGCCGTCGC
mouse	*S100a11 (sgRNA-1)*	CACCGTGTTAGGAACGGGGCATGG	AAACCCATGCCCCGTTCCTAACAC
mouse	*S100a11 (sgRNA-2)*	CACCGTGTTAGGAACGGGGCATGG	AAACCCATGCCCCGTTCCTAACAC
Molecular Cloning Primers			
Species	Gene	Forward (5'-3')	Reverse (5'-3')
human	*pSEB-3Flag-S100A11*	GGA AGATCT ACC ATGGCAAAAATCTCCAGCCCT	TGC GGATCC GGTCCGCTTCTGGGAAGGG
human	*pBu-3HA-AKT1*	TGG GGTACC ATG GGC AGCGACGTGGCTATTGTGA	TGA AGATCT CT GGCCGTGCCGCTGGCC
human	*pBu-3HA-ΔC (AKT1 1-151aa)*	TGG GGTACC ATG GGC AGCGACGTGGCTATTGTGA	TGA AGATCT CT CTCAAACTCGTTCATGGTCAC
human	*pBu-3HA-ΔN (AKT1 108-480aa)*	TGG GGTACC ATG GCTGACGGCCTCAAGAAGCAG	TGA AGATCT CT GGCCGTGCCGCTGGCC
human	*pBu-3HA-PCK1*	CGGGGTACCATGGGCCCTCCTCAGCTGCAAAACGGC	CTGGATATCCATCTGGCTTATTCTTTGCTTCAAG
human	*pBu-3HA-G309R*	GAAGGTTGAGTGCGTCAGGGATGACATTGCCT	AGGCAATGTCATCCCTGACGCACTCAACCTTC

Supplemental Table S2. Reagent or resource

REAGENT or RESOURCE	SOURCE	Identifier
**Antibodies**		
anti-PCK1	Bioworld	Cat# BS6870; RRID: AB_2895139
anti-H3K9me3	Abcam	Cat# ab8898; RRID: AB_306848
anti-H3K9me2	Abcam	Cat#ab176882; RRID: AB_2895140
anti-H3K9me1	Abcam	Cat#ab176880; RRID: AB_2751009
anti-H3K4me3	Cell signaling	Cat# 9751; RRID: AB_2616028
anti-H3K4me2	Cell signaling	Cat# 9725; RRID: AB_10205451

anti-H3K4me1	Cell signaling	Cat# 5326; RRID: AB_10695148
anti-H3K27me3	Cell signaling	Cat# 9733; RRID: AB_2616029
anti-H3K27me2	Cell signaling	Cat# 9728; RRID: AB_1281338
anti-H3K27me1	Bioworld	Cat# BS7235; RRID: AB_2934024
anti-H3K36me3	Cell signaling	Cat# 4909; RRID: AB_1950412
anti-H3K36me2	Cell signaling	Cat# 2901; RRID: AB _1030983
anti-H3K36me1	Cell signaling	Cat# 14111; RRID: AB _2798395
anti-MAT1A	Bioworld	Cat#BS8325; RRID: AB_2934025
anti-MTHFR	Bioworld	Cat#MB64078; RRID: AB_2934026
anti-MTR	Proteintech Group	Cat#25896-1-AP; RRID: AB_2880287
anti-MTHFD1	Proteintech Group	Cat#10794-1-AP; RRID: AB_2147391
anti-MTHFD2	Proteintech Group	Cat#12270-1-AP; RRID: AB_2147525
anti-SHMT1	Proteintech Group	Cat#14149-1-AP; RRID: AB_2239222
anti-SHMT2	Proteintech Group	Cat#11099-1-AP; RRID: AB_2188452
anti-m6A	Abcam	Cat#ab208577; RRID: AB_2916290
anti-G9A	Abcam	Cat# ab185050, RRID: AB_2792982
anti-EGLN3	Proteintech Group	Cat#18325-1-AP; RRID: AB_10640673
anti-SUV39H1	Cell signaling	Cat# D11B6; RRID: AB_10829612
anti-PHGDH	Proteintech Group	Cat#14719-1-AP; RRID: AB_2283938
anti-S100A11	Proteintech group	Cat#10237-1-AP; RRID: AB_2183478
anti-Flag	Sigma-Aldrich	Cat# F3165; RRID: AB_259529
anti-HA	Cell signaling	Cat# 3724; RRID: AB_1549585
anti-AKT1	Cell signaling	Cat#2938; RRID: AB_915788
anti-AKT	Bioworld	Cat# AP0059; RRID: AB_2797444
anti-p-AKT(Thr308)	Bioworld	Cat# AP0056; RRID: AB_2797442
anti-p-AKT(Ser473)	Bioworld	Cat# BS4007; RRID: AB_1662951
anti-N-Cadherin	Cell signaling	Cat# 13116; RRID: AB_2687616
anti-p21 Waf1/Cip1	Cell signaling	Cat# 2947; RRID: AB_823586
anti-MMP11	Bioworld	Cat# BS1230; RRID: AB_1663900
anti-H3	Sino Biological	Cat#100005-MM01; RRID: AB_2860034
anti-β-actin	ZSGB-BIO	Cat# TA-09; RRID: AB_2636897
Goat anti-rabbit, secondary	Abcam	Cat# ab6721; RRID: AB_955447
Goat anti-mouse, secondary	Abcam	Cat# ab6789; RRID: AB_955439
Goat anti-mouse /TRITC, secondary	ZSGB-BIO	Cat# ZF-0313; RRID: AB_2571577
Goat anti-mouse/ FITC, secondary	ZSGB-BIO	Cat# ZF-0312; RRID: AB_2716306
Goat anti-rabbit/ TRITC, secondary	ZSGB-BIO	Cat# ZF-0316; RRID: AB_2728778
Goat anti-rabbit/ FITC, secondary	ZSGB-BIO	Cat# ZF-0311; RRID: AB_2571576
**Chemicals**		
2×Taq PCR Green Mix	Dinguo	Cat#PER007

Carbon tetrachloride (CCl_4)	Macklin	Cat#C805332
Diethylnitrosamine (DEN)	Sigma	Cat#N0756
DMEM	HyClone	Cat#SH30243.01
DMEM	Gibco	Cat#A1443001
DMEM	Gibco	Cat#21013024
RPIM	BioAGRIO	Cat#LR1634-500
Lipofectamine 8000	Beyotime	Cat#C0533
Phospho(enol)pyruvic acid monopotassium salt	Sigma	Cat# P7127
Penicillin-Streptomycin Solution	HyClone	Cat#SV30010
Polybrene	Yeasen	Cat#40804ES76
Opti-MEM	Gibco	31985070
Suero fetal bovino esteril	Natocor	Cat# 12483020
Trypsin	Gibco	Cat#15050057
Trypsin-EDTA	Gibco	Cat#25200072
Matrigel basement membrane matrix	Corning	Cat#354234
serine	Sigma	Cat#S4500
glycine	Sigma	Cat#50046
methionine	Sigma	Cat#M9625
3PG	Sigma	Cat#P8877
SAM	Sigma	Cat#A7007
SAH	MCE	Cat#HY-19528
NCT503	Targetmol	Cat#T4213
(+)-Glucose solution	Gibco	Cat#A1443001
Sodium pyruvate solution	Sigma	Cat#S8636
Phenol red	Solarbio	Cat#P8460
L-cystine	MCE	Cat#HY-N0394
U- [^{13}C]- glutamine	Cambridge Isotope Laboratorics, Inc	Cat#CLM-1822-H-0.1
U- [^{13}C]- pyruvate	Cambridge Isotope Laboratorics, Inc	Cat#CLM-2440-0.1
U- [^{13}C]- methionine	Cambridge Isotope Laboratorics, Inc	Cat#CLM-893-H-0.05
**Critical Commercial Assays**		
BCA protein assay Kit	Dinguo	Cat#BCA02
Histostain™ - SP Kits	ZSGB-BIO	Cat#SPN-9001
DAB kit	ZSGB-BIO	Cat#ZLI-9019
DAPI	Roche	Cat#10236276001
Elivision™ plus Polyer HRP (Mouse/Rabbit) IHC Kit	Maxim	Cat#KIT-9901
Genomic DNA Purification Kit	Genloci	Cat#GP0155
PrimeScript® RT reagent Kit	Takara	Cat#RR047A

TRIzol™ Reagent	Invitrogen	Cat#15596026
TIANSeq mRNA Capture Kit	TIANGEN	Cat#1NR105
**Experimental Models: Cell Lines**		
HEK293	Lab stock	N/A
HEK293T	Lab stock	N/A
PLC/PRF/5	Lab stock	N/A
SK-Hep1	ATCC	HTB-52
SNU449	Lab stock	N/A
MHCC97H	Lab stock	N/A
Hep1-6	Lab stock	N/A
**Experimental Models: Organisms/Strains**		
BALB/c nude mice (male)	Experimental Animal Center of Chongqing Medical University	N/A
Pck1$^{(flox/flox)}$ C57 mice	EMMA:011950-UNC	the Mutant Mouse Resource & Research Centers
Cre$^{(+/+)}$ mice	Nanjing, China	Model Animal Research Center of Nanjing University
**Oligonucleotides**		
See Table S1 for sgRNA and qPCR primer sequences	IDT	N/A
**Recombinant DNA**		
Plasmid: pSEB-3Flag-S100A11	This paper	N/A
Plasmid: pBu-3HA-AKT1	This paper	N/A
Plasmid: pBu-3HA-ΔC (AKT1 1aa-151aa)	This paper	N/A
Plasmid: pBu-3HA-ΔN (AKT1 108aa-480aa)	This paper	N/A
Plasmid: pBu-3HA-PCK1	This paper	N/A
Plasmid: pBu-3HA-G309R	This paper	N/A
**Software and Algorithms**		
Graphpad Prism	GraphPad	https://www.graphpad.com
ImageJ	ImageJ Software	RRID: SCR_003070
Integrative Genomics Viewer	This paper	N/A
RNA-Seq	BioProject	PRJNA818729
ChIP-Seq	BioProject	PRJNA818729
RRBS	GEO	GSE221725
